# An Adaptive and Robust Test for Microbial Community Analysis

**DOI:** 10.3389/fgene.2022.846258

**Published:** 2022-05-19

**Authors:** Qingyu Chen, Shili Lin, Chi Song

**Affiliations:** ^1^ Division of Biostatistics, College of Public Health, The Ohio State University, Columbus, OH, United States; ^2^ Department of Statistics, College of Arts and Sciences, The Ohio State University, Columbus, OH, United States

**Keywords:** human microbiome, association test, community-level test, OTU-level test, adaptive combination of p-values

## Abstract

In microbiome studies, researchers measure the abundance of each operational taxon unit (OTU) and are often interested in testing the association between the microbiota and the clinical outcome while conditional on certain covariates. Two types of approaches exists for this testing purpose: the OTU-level tests that assess the association between each OTU and the outcome, and the community-level tests that examine the microbial community all together. It is of considerable interest to develop methods that enjoy both the flexibility of OTU-level tests and the biological relevance of community-level tests. We proposed MiAF, a method that adaptively combines *p*-values from the OTU-level tests to construct a community-level test. By borrowing the flexibility of OTU-level tests, the proposed method has great potential to generate a series of community-level tests that suit a range of different microbiome profiles, while achieving the desirable high statistical power of community-level testing methods. Using simulation study and real data applications in a smoker throat microbiome study and a HIV patient stool microbiome study, we demonstrated that MiAF has comparable or better power than methods that are specifically designed for community-level tests. The proposed method also provides a natural heuristic taxa selection.

## 1 Introduction

Investigating the function of the microbiome in human health has become a burgeoning study field in recent years, which is attributed to the advent of new technologies for profiling complex microbial communities by 16 S rRNA gene sequencing (Lasken, 2012) or shotgun metagenomic sequencing (Hasan et al., 2014). Various microbial communities live throughout the human body and are associated with several diseases, such as colorectal cancer (Ahn et al., 2013), inflammatory bowel disease (Kostic et al., 2014) and obesity (Ley, 2010). Understanding the association between the microbiome and human disease may push back the frontiers of medical treatment.

Although the shotgun metagenomic sequencing enjoys higher resolution of taxonomic identification (Hasan et al., 2014), the reduced cost of 16 S rRNA gene sequencing makes it a more commonly used technology for microbiome studies to date. Using standard pipelines, 16 S sequences are clustered based on a prespecified similarity threshold (typically 97%) into operational taxonomic units (OTUs), each of which represents a taxonomic unit at a certain taxonomic rank, such as order, family, or genus (Nguyen et al., 2016). We note that some pipelines such as DADA2 (Callahan et al., 2016) and Deblur (Amir et al., 2017) generate amplicon sequence variants (ASVs) instead of traditional OTUs. ASVs can be viewed as OTUs with the exact same sequences, and are sometimes referred as 100% OTUs. Because the analysis methods discussed here can be applied to both OTUs and ASVs, we will not differentiate them and refer to both as OTUs in the rest of this paper. Since the initiation of Human Microbiome Project (Turnbaugh et al., 2007) in 2007, researchers have developed a variety of statistical methods to detect the possible association between microbiome diversity and an outcome of interest, such as a disease status.

There are two general categories of approaches for detecting associations—OTU-level methods and community-level methods. OTU-level methods test whether each individual OTU is associated with the outcome, while community-level methods test whether the microbial community in its entirety is associated with the outcome. Typically, the OTU-level methods test the association between a clinical outcome and the abundance of each OTU as a univariate covariate one-by-one. This univariate approach allows the development of many sophisticated OTU-level methods that can carefully accommodate the discrete and sparse nature of OTU-level abundance data. For example, QIIME (Caporaso et al., 2010), as a comprehensive pipeline, have the capability of performing OTU differential abundance tests using metagenomeSeq zero-inflated Gaussian (Paulson et al., 2013) and DESeq2 negative binomial Wald test (Love et al., 2014). The former developed a zero-inflated Gaussian distribution mixture model to avoid biases due to undersampling of the microbial community, while implementing a normalization method to deal with uneven sequencing depth. The later adapted the negative binomial model that has been popular in gene differential expression study to analyze microbiome data. In addition, QIIME also contains several classic statistical tests, such as ANOVA, Kruskal-Wallis, G-test, Mann-Whitney test, as well as the parametric and nonparametric t-test.

In practice, it is frequently more biologically relevant to perform community-level analysis, which jointly tests the association between a clinical outcome and a microbial community as a whole. These methods are often based on alpha diversity or beta diversity. Alpha diversity characterizes the complexity of the microbial community within each sample. Among them, the Inverse Simpson Diversity (Simpson, 1949), Shannon Indexes (Shannon, 1948) and Faith’s phylogenetic diversity that incorporates phylogenetic relationships (Faith, 1992) are some of the most popular choices. After summarizing the complexity of the microbial community into a single alpha diversity metric, univariate methods such as regression models can be applied to detect the possible association between the alpha diversity and the clinical outcome. Adaptive microbiome *α*-diversity-based association analysis (aMiAD) (Koh, 2018) used the minimum *p*-value from association analyses based on different alpha diversity metrics as its test statistic, and assessed the *p*-value of the proposed test *via* a residual-based permutation method. Beta diversity, on the other hand, measures the distance or dissimilarity between each pair of biological samples. For example, Bray-Curtis dissimilarity measures the differences between two microbial communities by quantifying the non-overlapping OTU abundances (Bray and Curtis, 1957). Jaccard distance can be viewed as an “unweighted” version of Bray-Curtis dissimilarity, since it only relies on the presence or absence of OTUs without taking abundance information into account (Jaccard, 1901, Jaccard, 1912). Among many available distance metrics, the UniFrac distance incorporating phylogenetic information is one of the most popular metrics (Lozupone et al., 2007). It calculates the fraction of sums of branch lengths with their corresponding taxa only in one sample to both samples. Both weighted and unweighted versions of UniFrac are commonly used in microbial ecology, where the former accounts for abundance information of the taxa, while the latter only considers their presence or absence. Moreover, generalized UniFrac distances were proposed as a series of distance metrics—from unweighted to weighted UniFrac by assigning different weights on the branches (Chen et al., 2012). Based on the beta diversity or a distance metric, various community-level association testing methods have been proposed. Permutational Multivariate Analysis of Variance (PERMANOVA) (McArdle and Anderson, 2001), one of the pioneer community-level tests, is a non-parametric method that fits multivariate models for microbial community data to test whether the samples significantly differ across a categorical factor. It bears some resemblance to ANOVA but operates on a dissimilarity matrix and assesses *p*-values based on permutation. However, PERMANOVA usually adopts only one of the many available distance metrics with no confounder adjustment and cannot easily accommodate continuous traits (unless categorized arbitrarily). Microbiome Regression-based Kernel Association Test (MiRKAT) (Zhao et al., 2015), a more comprehensive method, was proposed to extend the outcome of interest to the continuous case. The phylogenetic dissimilarity matrix is transformed into a kernel matrix which measures the similarity of microbial communities between samples. MiRKAT regresses the clinical outcome on this semiparametric kernel machine while adjusting for potential confounders. It should be noted that MiRKAT is equivalent to PERMANOVA when no covariates are included. Besides, MiRKAT can combine multiple distance metrics by selecting the one that generates the smallest *p*-value.

Although OTU-level methods and community-level methods tackle the association testing problem from different angels, they are in fact related to each other. The statement that the microbial community is associated with the clinical outcome is equivalent to that at least one of the OTUs differs across the outcome status. Therefore, theoretically, the results of all the OTU-level tests can be summarized across the observed taxon units to draw a community-level conclusion about whether the microbial community is associated with the clinical outcome. Considering the vast availability of univariate models for different study designs that can be directly applied to OTU-level analysis, as well as the sophisticated OTU-level methods that accommodate unique aspects of microbiome data, it would be beneficial to combine them into community-level tests.

However, simply putting all OTU-level tests together without proper weighting or OTU selection will suffer from power loss, because not all OTUs may be associated with the outcome, and as thus, a naive combination may accumulate noises that eventually surpass association signals. Moreover, the number or proportion of OTUs that are not associated with the outcome is often unknown in practice. In contrast, adaptively and wisely assigning weights to the taxon units according to their importance is a key to achieving greater statistical power. Some efforts have already been put into this area. For example, adaptive Microbiome-based Sum of Powered Score (aMiSPU) test (Wu et al., 2016) extended the aSPU test (Pan et al., 2014) to accommodate unique features of microbial data. This method adaptively combines the score statistics for two versions of generalized taxon proportions and resembles MiRKAT with weighted and unweighted UniFrac kernel. OMiAT (Koh et al., 2017) combines aSPU and MiRKAT by taking the minimum *p*-value from all the score tests of the two methods. aSPU used in OMiAT implements on standard compositional microbial data without incorporating phylogenetic information. However, the requirement of score statistic for taxon units in aMiSPU and aSPU may limit their applicability to different study designs where the score statistics may not be readily available. This requirement also makes aMiSPU and OMiAT inflexible to combine more sophisticated OTU-level testing methods that are specifically designed for microbiome data. Compared to score statistic, *p*-value is a more universally available statistic in OTU-level association tests, thus making it a more suitable target to combine, for the sake of flexibility. MiHC (Koh and Zhao, 2020), adapted from higher criticism test which aims to detect highly sparse signals, was tailored to accommodate different sparsity levels and incorporate phylogenetic information. It was more powerful for sparse microbial association signals than abundant ones. In this paper, inspired by Adaptive Fisher (AF) method (Song et al., 2016), we propose a *p*-value combination approach, Microbiome Adaptive Fisher method (MiAF), to aggregate *p*-values of OTU-level tests into a novel community-level association test. It should be noted that the focus of MiAF is to test whether the OTU community is associated with the outcome, instead of estimating the parameters of the association model. We compare the performance of MiAF to methods specifically designed for detecting community-level associations, and demonstrates comparable or better power for MiAF. We also discuss the potential of MiAF as a general *p*-value combination framework for microbial community-level tests under various study designs.

## 2 Materials and Methods

### 2.1 Statistical Model and OTU-Level Tests

**FIGURE 1:**
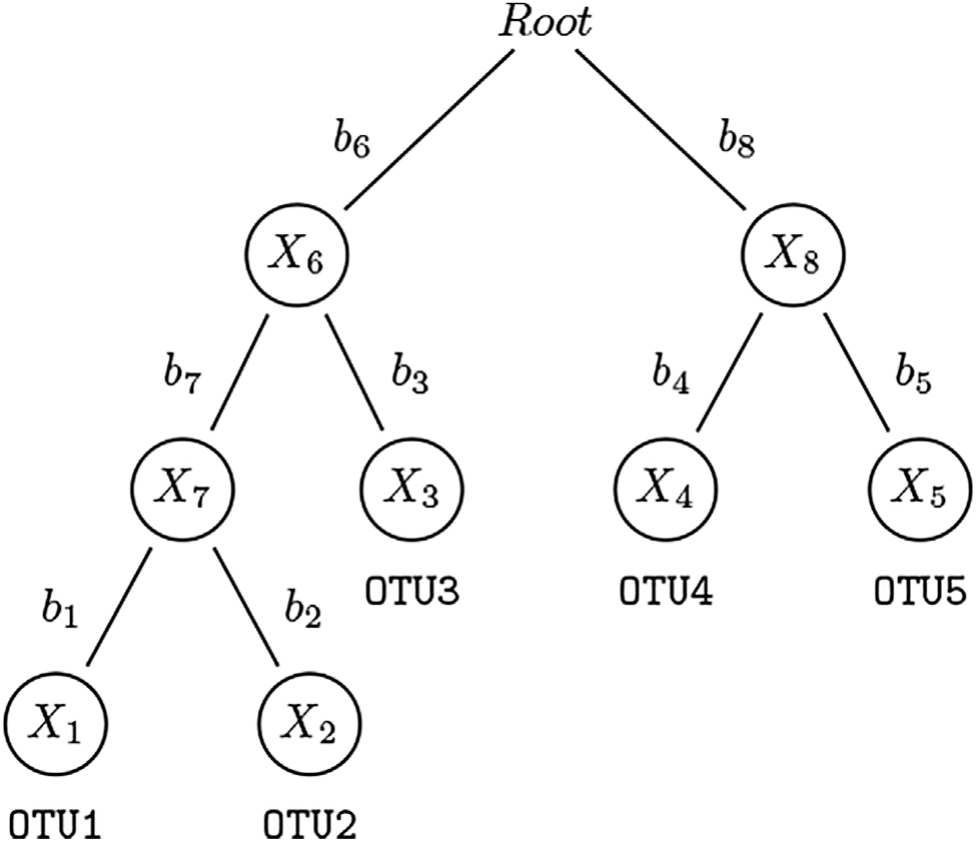
An example of a rooted phylogenetic tree. This is a simple rooted phylogenetic tree containing 5 leaf nodes and 4 internal nodes. The counts for leaf nodes—OTU1–5 — can be obtained *via* standard pipelines. We assign the counts to internal nodes by summing up the counts of their children and refer to them also as “OTUs”: For three of the four abundance representations used in this paper, OTU7 = OTU1 + OTU2, OTU6 = OTU7 + OTU3, and OTU8 = OTU4 + OTU5. We construct the extended OTU matrix containing all nodes (except the root) in this phylogenetic tree.

Suppose *n* subjects are observed and their microbial communities are profiled. For the *i*th subject, *Y*_*i*_ denotes the outcome of interest which can be binary or continuous, and ***Z***_*i*_ = (*Z*_*i*1_, *…*, *Z*_*ic*_) denotes *c* covariates such as age and gender that are potentially associated with both the clinical outcome and microbial community, which we need to adjust for as potential confounders. We construct an “extended” OTU table containing all nodes (terminal and internal) in the phylogenetic tree. Let ***X***_*i*_ = (*X*_*i*1_, *…*, *X*_*im*_) be the counts of “extended” OTUs which consist of both leaf nodes and internal nodes (except for root node) for subject *i*, where *m* is the total number of “extended” OTUs. The count of an internal node is derived by summing up all the counts of the leaf node OTUs belonging to this taxon (see Figure 1 for an illustration). Note that our method is not limited to bifurcating phylogenetic trees, it is applicable to multifurcating trees. The relative abundance of extended OTU *k*, *k* = 1, *…*, *m*, in subject *i*, *i* = 1, *…*, *n*, is $A_{ik}=X_{ik}/\sum_{j=1}^{q}X_{ij}$ , where *q* is the number of leaf nodes, and the *X*_*ij*_’s are arranged such that the first *q* entries in ***X***_*i*_ are the leaf nodes in the same order for all individuals.

OTU abundance varies greatly in a microbial community. Some microbes are dominant, but most are rare. In practice, the underlying association patterns are unknown a priori. We do not have the knowledge of the characteristics of the truly associated OTUs nor their phylogenetic relationships that are captured by phylogenetic trees. Therefore, we incline to integrate the abundance information and phylogenic relationships adaptively to achieve a robust test under diverse underlying situations. When the associated OTUs are indeed phylogenetically related, incorporating phylogenetic information may boost the performance of an association analysis to a great extent. To accommodate such a situation, we define unweighted and weighted taxon proportions as $M_{ik}^{u}=I\left(A_{ik}>0\right)$ and $M_{ik}^{w}=A_{ik}$ respectively for “extended” OTU k, *k* = 1, *…*, *m*. The unweighted taxon proportion only considers the presence or absence of an OTU, whereas the weighted one takes the magnitude of the abundance information into account. Inspired by the generalized UniFrac distance metric (Chen et al., 2012), we also define a square-root transformed taxon proportion to attenuate the contribution by highly abundant OTUs as $M_{ik}^{.5}={A_{ik}}^{0.5}$ .

We also consider a taxon proportion restricted to leaf nodes only for situations where the associated OTUs are not phylogenetically related, since incorporating phylogenetic information in this scenario may adversely affect testing performance. That is, we only include the weighted taxon proportion of leaf nodes in original OTU table defined as $M_{ik}^{a}=A_{ik}$ , *k* = 1, *…*, *q*.

We use the following generalized linear model to depict the association between the compositions of microbes in a community and the health outcome taking confounding covariates into consideration:

$$h\left(E\left[Y_{i}\right]\right)=\alpha_{0}+Z_{i}\alpha +\overset{d}{\underset{k=1}{\sum }}M_{ik}\beta_{k},$$ (1)

where $\alpha ={\left(\alpha_{1},\ldots \alpha_{c}\right)}^{⊤}$ represents the effects of the *c* covariates, $\beta ={\left(\beta_{1},\ldots \beta_{d}\right)}^{⊤}$ are the effects of the OTUs, and *M*_*ik*_ can be any of the four abundance representations defined above ( $M_{ik}^{u}$ , $M_{ik}^{w}$ , $M_{ik}^{.5}$ or $M_{ik}^{a}$ ); thus *d* = *q* for $M_{ik}^{a}$ and *d* = *m* for the other three measures. Finally, *h* (⋅) is the link function, which is the logit function for binary outcomes or the identity function for continuous outcomes.

We are interested in determining whether there is an association between the outcome of interest and any OTU, which is equivalent to testing the following hypotheses:

$$H_{0}:\beta =0\text{ vs. }H_{1}:\beta \neq 0.$$

The score statistics ***U*** = (*U*_1_, *…* , *U*_*d*_) for ***β*** can be calculated as $U=\sum_{i=1}^{n}\left(Y_{i}-{\hat{\mu }}_{i}\right)\left(M_{i}-\hat{M_{i}}\right)$ , where ${\hat{\mu }}_{i}$ is the expectation of *Y*_*i*_ under *H*_0_, and $\hat{M_{i}}=\left(\hat{M_{i1}},\ldots \hat{M_{id}}\right)$ are the fitted values of ***M***_*i*_ by regressing ***M***_⋅*k*_ = (*M*_1*k*_, *M*_2*k*_, *…* , *M*_*nk*_), for each *k* = 1, *…* , *d*, separately on the covariates ***Z***. Under *H*_0_, ***U*** ∼ *N* (***0***, ***V***), where ***V*** is the corresponding Fisher information matrix. Then the marginal OTU-level *p*-values ***p*** = (*p*_1_, … , *p*_*d*_) for ***β*** can be obtained based on $\tilde{U}=\left({\tilde{U}}_{1},\ldots ,{\tilde{U}}_{d}\right)$ , where ${\tilde{U}}_{k}=U_{k}/V_{kk}$ and *V*_*kk*_ is the *k*th diagonal element of ***V***. We noted that in this paper, we choose to combine the one-sided *p*-values (i.e., $p_{k}^{l}=\Phi \left({\tilde{U}}_{k}\right)$ for the lower-tail and $p_{k}^{u}=1-\Phi \left({\tilde{U}}_{k}\right)$ for the upper-tail), because they account for the directionality of effects and can help boost statistical power when many OTUs have effects of the same direction. We also note that in rare situations where *V*_*kk*_ = 0 for some OTU *k*, we remove these OTUs from any subsequent analysis.

### 2.2 Combining P-Values from OTU-Level Tests

After getting *p*-values for all the OTUs (either the “extended” set or the original set), we combine them as follows. Let

$$R_{k}=-\log p_{k},$$ (2)

where *p*_*k*_ is the *p*-value for testing OTU *k*, which can be $p_{k}^{l}$ or $p_{k}^{u}$ as defined above, for a particular abundance representation ***M*** (***M*** ^*u*^, ***M*** ^*w*^, ***M*** ^.5^ or ***M*** ^*a*^). Since the taxa in the phylogenetic tree represent different classification levels and the abundance dispersion of different OTUs varies drastically, not all OTUs in a microbial community contribute, let alone contribute equally, to the clinical outcome of interest. Therefore, assigning different weights to OTUs according to their potential importance may enhance the statistical power of the association test. In our method, when including internal nodes, i.e., using $M_{ik}^{u}$ , $M_{ik}^{w}$ , or $M_{ik}^{.5}$ , we use a UniFrac-like weight

$$\omega_{k}=\text{SD}\left(M_{\cdot k}\right)\times b_{k},\;\;\;\;k=1,\ldots ,m,$$ (3)

where *b*_*k*_ is the length of the branch that leads to the *k*th OTU in the phylogenetic tree, and SD (⋅) stands for standard deviation. Our choice of weights takes into account both the dispersion of OTUs and their positions in the phylogenetic tree, and it is the same as that used in MiSPU and MiRKAT with UniFrac kernels if these methods are viewed as combining standardized score statistics. For $M_{ik}^{a}$ , since only leaf nodes are considered, the branch length is no longer relevant; thus, we use

$$\omega_{k}=\text{SD}\left(M_{\cdot k}\right),\;\;\;\;k=1,\ldots ,q.$$ (4)

Given the weights ***ω*** = (*ω*_1_, *…*, *ω*_*d*_) for all *d* OTUs, we can calculate

$$W_{k}=\omega_{k}R_{k}.$$ (5)

Then we sort *W*_1_, *…*, *W*_*d*_ in descending order, such that *W*_(1)_ ≥⋯ ≥ *W*_(*d*)_. Let ***S*** = (*S*_1_, *…*, *S*_*d*_) be the partial sum of *W*_(1)_, *…*, *W*_(*d*)_, i.e.

$$S_{k}=\overset{k}{\underset{l=1}{\sum }}W_{\left(l\right)}.$$ (6)

For each *S*_*k*_, its *p*-value can be defined as $P_{s_{k}}=\mathrm{Pr}\left(S_{k}\geq s_{k}\right)$ , where *s*_*k*_ is the observed value of *S*_*k*_, for *k* = 1, *…* , *d*. This leads to our proposed AF statistic

$$T_{AF}=\underset{1\leq k\leq d}{\min}P_{s_{k}},$$ (7)

The minimizer in Equation 7 casts some light on the associated taxa, thus, we provide a heuristic taxon selection procedure. Suppose $h={argmin}_{1\leq k\leq d}P_{s_{k}}$ , we select *h* taxa corresponding to the *h* largest *W*_*k*_s as associated with the outcome. However, we caution against over-interpreting the taxon selection results, which we will further explore in Section 3.1.2 and Section 3.2.

### 2.3 Assessing Statistical Significance by Permutation

Since the asymptotic distributions of *S*_*k*_ and *T*_*AF*_ are intractable when the OTU abundances are correlated, we propose to carry out the following permutation algorithm to access the null distribution of *T*_*AF*_ and estimate its corresponding *p*-value.

Step 1. Regress each OTU column of ***M***, ***M***_⋅*k*_, iteratively on the covariates ***Z*** to obtain the fitted OTU matrix $\hat{M}$ and the corresponding residual matrix $\tilde{M}=M-\hat{M}=\left\{\tilde{M_{ij}}\right\}$ . Calculate marginal *p*-values ***p*** for the OTUs according to model Eq. 1 using $\tilde{M}$ as ***M***. Set ***p*** ^(0)^ = ***p***.

Step 2. Permute rows of $\tilde{M}$ for a large number of times, *B*, to get a set of permuted residual matrices $\left\{{\tilde{M}}^{\left(1\right)},\ldots ,{\tilde{M}}^{\left(B\right)}\right\}$ . Obtain the permutation set of *p*-values {***p*** ^(1)^, *…*, ***p*** ^(*B*)^}, by refitting the regression model with the permuted residuals for *b* = 1, *…*, *B*.

Step 3. Follow Equations 2–6 to obtain $S^{\left(b\right)}=\left(S_{1}^{\left(b\right)},\ldots ,S_{d}^{\left(b\right)}\right)$ , for *b* = 0, 1, *…*, *B*, where ***S*** ^(0)^, corresponding to ***p*** ^(0)^, denoting the statistic based on the original data.

Step 4. For each *b* = 0, 1, …, *B* and *k* = 1, *…*, *d*, calculate

$$P_{S_{k}}^{\left(b\right)}\approx \frac{1}{B+1}\overset{B}{\underset{b^{*}=0}{\sum }}I\left\{S_{k}^{\left(b^{*}\right)}\geq S_{k}^{\left(b\right)}\right\}.$$

Then we can get the observed and permuted AF statistics $T_{AF}^{\left(b\right)}=\min_{1\leq k\leq d}P_{S_{k}}^{\left(b\right)}$ , for *b* = 0, 1, *…*, *B*.

Step 5. The *p*-value $P_{AF}^{\left(b\right)}$ of the AF statistic $T_{AF}^{\left(b\right)}$ can be approximated by

$$P_{AF}^{\left(b\right)}=\text{Pr}\left\{T_{AF}\leq T_{AF}^{\left(b\right)}\right\}\approx \frac{1}{B+1}\overset{B}{\underset{b^{*}=0}{\sum }}1\left\{T_{AF}^{\left(b^{*}\right)}\leq T_{AF}^{\left(b\right)}\right\},$$

where *b* = 0, 1, …, *B*.

Note that in Step 1 and 2, we permute the residuals of regression ***M***_⋅*k*_ on ***Z*** and fit a generalized linear model using the permuted residuals, which preserves the correlation among covariates ***Z*** and abundance representation ***M*** even after permutation. We also noted that we used index *b* = 0 to denote the statistics calculated from the original observed data. Therefore $P_{AF}^{\left(0\right)}$ is the final *p*-value of our proposed AF statistic if there is only one list of OTU-level *p*-values ***p*** = (*p*_1_, … , *p*_*d*_) to combine, e.g., only ***p*** ^*l*^ using ***M*** ^*u*^. Besides, we also calculated $P_{AF}^{\left(b\right)}$ for *b* = 1, …, *B*, which are *B* permutations of the AF *p*-value. These permutations can be further used to combine the results of multiple AF *p*-values generated by combining *p*-values from our multiple OTU abundance representations, which we discuss next.

### 2.4 Combining Multiple AF Tests

In the method described in the previous subsections, there are multiple variations or factors that can affect the performance of the test under different scenarios, including the choice of OTU-level tests, the transformation from relative abundance, ***A***, to an abundance representation, ***M***, the usage of one-sided or two-sided *p*-values, and the weights used in the combination step. Therefore, to construct a statistical test that is robust under various scenarios, it is often desirable to combine the results from multiple tests based on different parameter choices. We therefore, propose to combine the results of multiple AF tests with different parameter selections to form a unified test.

The *p*-value combination approach that we described previously in Section 2.2 can be viewed as a general method for combining multiple *p*-values with or without weights, as long as we can obtain a permuted sample while preserving the correlation among them. We define operation *AF*{***p*** [; ***ω***]} as the procedure that combined a *p*-value vector ***p*** with optional weight vector ***ω***, which defaults to ones when omitted. By using this *AF* operator, we can redefine our MiAF method that combines results from different choices of OTU-level test *p*-values, weights, and abundance representations. For illustration purpose, in the rest of our paper, we combine results from lower- and upper-tail *p*-values using the unweighted (***M*** ^*u*^), weighted (***M*** ^*w*^), square-root (***M*** ^.5^) abundance representations for “extended” OTUs and their corresponding weights as defined above, as well as the abundance representations for leaf nodes only (***M*** ^*a*^) and its corresponding weights. Specifically, ***p*** ^*ul*^ and ***p*** ^*uu*^ denote the lower- and upper-tail *p*-values of the OTU-level tests using ***M*** ^*u*^. Similarly, we use ***p*** ^*wl*^ and ***p*** ^*wu*^ for ***M*** ^*w*^, ***p*** ^.5*l*^ and ***p*** ^.5*u*^ for ***M*** ^.5^, and ***p*** ^*al*^ and ***p*** ^*au*^ for ***M*** ^*a*^.

With the associated weights denoted as ***ω*** ^*a*^, ***ω*** ^*w*^, ***ω*** ^.5^ and ***ω*** ^*a*^, respectively, we can obtain the *p*-value for each of the eight community-level MiAF tests by combining the corresponding OTU-level *p*-value vectors and the corresponding weight vectors using the *AF* operator defined above; details are given in the 5^*th*^ and 6^*th*^ columns of Table 1. The two one-sided community-level tests are then combined to form a two-sided test, again using the *AF* operator, for each of the four abundance measure tests (column 7 of Table 1). Our eventual test statistic, MiAF, combines the unweighted UniFrac-like test *p*-value $P_{MiAF_{u}}$ , the weighted UniFrac-like test *p*-value $P_{MiAF_{w}}$ , the generalized UniFrac-like test *p*-value $P_{MiAF_{.5}}$ and the leaf-nodes-only test *p*-value $P_{MiAF_{a}}$ , again using *AF* operator (last row of Table 1). We declare that the microbial community is significantly associated with the clinical outcome if *P*_*MiAF*_ is smaller than a prespecified significance level *α*.

**TABLE 1:** MiAF implementation algorithm.

Tests	Abundance measure	Relationship to *A*	Phylogenetic information	Single measure		Combine multiple measures
				Lower-Tail	Upper-Tail	
MiAF_*u*_	***M*** ^*u*^	${M_{ik}^{u}=I\left(A_{ik}>0\right)}^{*}$	✓	*P* ^*ul*^ = *AF*{***p*** ^*ul*^; ***ω*** ^*u*^}	*P* ^*uu*^ = *AF*{***p*** ^*uu*^; ***ω*** ^*u*^}	$P_{MiAF_{u}}=AF\left\{{\left(P^{ul},P^{uu}\right)}^{T}\right\}$
MiAF_*w*_	***M*** ^*w*^	${M_{ik}^{w}=A_{ik}}^{*}$	✓	*P* ^*wl*^ = *AF*{***p*** ^*wl*^; ***ω*** ^*w*^}	*P* ^*wu*^ = *AF*{***p*** ^*wu*^; ***ω*** ^*w*^}	$P_{MiAF_{w}}=AF\left\{{\left(P^{wl},P^{wu}\right)}^{T}\right\}$
MiAF_.5_	***M*** ^.5^	${M_{ik}^{.5}={A_{ik}}^{.5}}^{*}$	✓	*P* ^.5*l*^ = *AF*{***p*** ^.5*l*^; ***ω*** ^.5^}	*P* ^.5*u*^ = *AF*{***p*** ^.5*u*^; ***ω*** ^.5^}	$P_{MiAF_{.5}}=AF\left\{{\left(P^{.5l},P^{.5u}\right)}^{T}\right\}$
MiAF_*a*_	***M*** ^*a*^	${M_{ik}^{a}=A_{ik}}^{†}$	✗	*P* ^*al*^ = *AF*{***p*** ^*al*^; ***ω*** ^*a*^}	*P* ^*au*^ = *AF*{***p*** ^*au*^; ***ω*** ^*a*^}	$P_{MiAF_{a}}=AF\left\{{\left(P^{al},P^{au}\right)}^{T}\right\}$
MiAF	—	—	—	—	—	*P*_*MiAF*_ = $AF\left\{{\left(P_{MiAF_{u}},P_{MiAF_{w}},P_{MiAF_{.5}},P_{MiAF_{a}}\right)}^{T}\right\}$

^*^ *k* = 1, *…*, *m*.

^†^ *k* = 1, *…*, *q*.

## 3 Results

### 3.1 Simulation Study

#### 3.1.1 Simulation Strategy

We conducted simulation studies to investigate whether MiAF correctly controls type I error and to evaluate the performance of MiAF in a wide range of scenarios. We generated unobvserved absolute abundances and read counts of OTUs using the R package SparseDOSSA2 which can parameterize real microbial profiles and then simulate new profiles based on the estimated parameters (Ma et al., 2021). SparseDOSSA2 depicts the unobserved absolute abundance *via* a Gaussian copula model with zero-inflated log normal marginal distributions. To address the identifiability issue, it imposes *L*_1_ penalization on the correlation matrix. Using SparseDOSSA2 package, we first parameterized a real upper-respiratory-tract microbiome data set consisting of 856 OTUs and 60 samples (Charlson et al., 2010). The penalizing tuning parameter was chosen to be 0.1 since it achieved the largest likelihood among {0.1, 0.2, *…*, 1}. 616 OTUs remained after discarding the OTUs with only one non-zero count across the samples. Then the microbial community profiles for 616 OTUs and 100 samples, including unobserved absolute abundance and read counts, based on the estimated parameters were simulated. We denoted the simulated absolute abundance matrix by ***X***, where *X*_*ij*_ was the absolute abundance of OTU *j* in sample *i*.

To evaluate our method, we implemented three simulation scenarios where the OTUs were divided into different clusters and related to both binary and continuous outcomes in different ways. The clustering on OTUs was based on partitioning around medoids (Kaufman and Rousseeuw, 1990) with cophenetic distance (Sokal and Rohlf, 1962). We chose three cluster numbers: 10, 22 and 29, corresponding to the first three local maxima of the mean silhouette values shown in Supplementary Figure S1 of Supplementary Material. Under scenario 1, the 616 OTUs were grouped into 22 clusters. The abundance varied greatly among these 22 clusters. In order to test our new method in broader circumstances, we performed the simulation analysis assuming that the outcome is truly associated with each cluster of OTUs iteratively instead of evaluating the performance on only a few clusters. The binary outcome *Y*_*i*_ for sample *i*, *i* = 1, *…*, 100, was simulated based on model

$$\text{logit}\left(E\left(Y_{i}|X_{i},Z_{i}\right)\right)=0.5\;\text{scale}\left(Z_{i1}+Z_{i2}\right)+\beta \;\text{scale}\left(\underset{j\in C}{\sum }X_{ij}\right).$$ (8)

We simulated continuous outcomes under the model

$$Y_{i}=0.5\;\text{scale}\left(Z_{i1}+Z_{i2}\right)+\beta \;\text{scale}\left(\underset{j\in C}{\sum }X_{ij}\right)+\epsilon_{i},$$ (9)

where *ϵ*_*i*_ ∼ *N*(0, 1). For both binary and continuous outcomes, *Z*_*i*1_ and *Z*_*i*2_ were covariates, and *C* was the set of OTUs that belong to a selected cluster. The scale(⋅) function standardizes the sample mean to 0 and standard deviation to 1. *Z*_*i*1_ was drawn from a Bernoulli distribution with success probability 0.5 independently. For *Z*_*i*2_, we consider two situations where *Z*_*i*2_ and the abundance of the microbial community ***X***_*i*⋅_ are either independent or correlated. In the independent case, *Z*_*i*2_ was generated from standard normal distribution *N* (0, 1), and the effect size *β* was set as 0.6, 0.8, 1.2, 1.6 and 2 for binary outcomes, and 0.2, 0.4, 0.6, 0.8 and 1 for continuous outcomes to mimic different levels of association strength between the OTUs and the clinical outcome. In the correlated case, we let *Z*_*i*2_ = scale(*∑*_*j*∈*C*_ *X*_*ij*_) + *τ*, where *τ* ∼ *N*(0, 1) and the effect size *β* was set to be twice as large as the corresponding value in the independent case, in order to show a clearer difference among the methods compared.

Under scenario 2, we divided the 616 OTUs into 10 clusters and simulated the data on all clusters following the same settings. For scenario 3, all OTUs were divided into 29 clusters following the same procedure.

Under all three simulation scenarios, the performance of MiAF was compared to MiRKAT, aMiSPU, OMiAT, aMiAD and MiHC. We did not include PERMANOVA because it is essentially equivalent to MiRKAT without covariates (Zhao et al., 2015). aMiSPU combines unweighted and weighted UniFrac versions of test. MiRKAT combines four kernels, including the unweighted and weighted UniFrac, a generalized UniFrac with tuning parameter at 0.5, and the Bray-Curtis. MiAF combines the unweighted, weighted, generalized UniFrac-like and the leaf-nodes-only test *p*-values. We used default setting of OMiAT, which includes all the kernels in MiRKAT but with an addition of the Jaccard distance. aMiAD combines six alpha diversity metrics as its default setting, which includes Richness, Shannon, Simpson, phylogenetic diversity (PD), phylogenetic entropy (PE) (Allen et al., 2009) and phylogenetic quadratic entropy (PQE) (Rao, 1982). MiHC combines the unweighted higher criticism test, weighted higher criticism test and Simes test, and the candidate set for both higher criticism tests to modulate low sparsity level was set as {1, 3, 5, 7, 9}. We set the significance level to be 0.05 for each test. When evaluating the type I error under all the simulation scenarios, we simulated data according to model (Eqs 8 and 9) by setting *β* = 0. We set the number of permutation for all five methods as 10,000 to assess their ability for correct control of type I error. When comparing power, the number of permutation was set to be 1,000. All simulation results were based on 1,000 independent replicates.

We investigated the performance of the proposed heuristic taxa selection procedure when setting *β* > 0 in the model (Eqs 8 and 9). Although only tip nodes were explicitly assumed to be associated with the outcome in the simulation setting, we also viewed the internal nodes as associated taxa if any of their descendants was associated. Since the outcome were generated to be positively correlated with the abundances of OTUs within the associated cluster, we recorded the number of every taxon being selected from upper-tail *p*-values in the 1,000 independent replicates.

#### 3.1.2 Simulation Results

**FIGURE 2:**
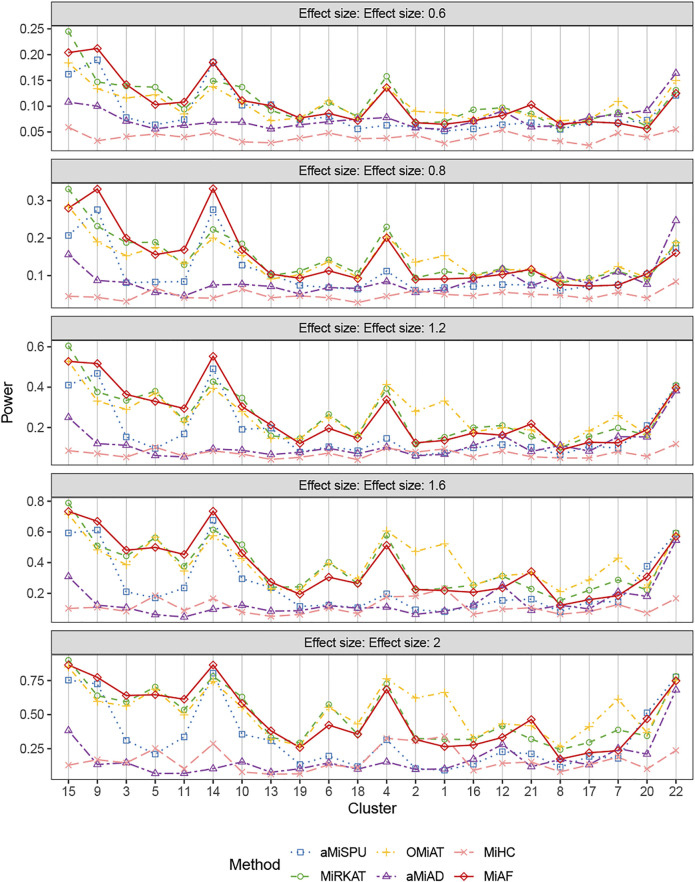
Power comparison for binary outcomes under the independent case of scenario 1. A total of 616 OTUs were divided into 22 clusters. The covariates *Z*_*i*2_ and OTUs ***X***_***i⋅***_ were independent. The effect size was set as 0.6, 0.8, 1.2, 1.6 and 2. The 22 clusters were sorted by the sum of estimated mean absolute abundance of the OTUs within the cluster that was truly associated from the greatest to the least.

Figure 2 shows the statistical power for binary outcomes under scenario 1, where 616 OTUs were partitioned into 22 clusters, and when both covariates *Z*_*i*1_ and *Z*_*i*2_ are independent of the microbial community. The cluster size and mean absolute abundance varies greatly among 22 clusters (see details in Supplementary Table S1B of Supplementary Material), covering different underlying association patterns. We evaluated the performance of all the methods under situations where each phylogenetic cluster of OTUs was set to be associated with the binary outcome successively. The power of the six methods was plotted against clusters sorted by the sum of estimated mean absolute abundance of OTUs within the cluster that was truly associated from the greatest to the least, representing the total strength of signals. As expected, for each associated cluster community, the statistical power increased as the effect size *β* increased. For aMiSPU, MiRKAT and MiAF, the performance of the unweighted version of tests was outperformed by the weighted version of tests in the majority of the clusters, with exceptions of clusters 7, 12, 16, 20 and 22. Another observation for all methods was that the combined tests lose only a little power compared to the best one of their corresponding component tests, which justifies the use of a combined or optimal test to draw a unified conclusions from multiple parameter choices. Therefore, we focused on comparing the performance of the combined version of the six tests in the rest of this section. When the total sum of estimated mean absolute abundance of OTUs within the associated clusters was relatively large (around top 40% of the sum of absolute abundance of associated OTUs among the 22 clusters), MiAF either outperformed the other five methods or was commensurate with the best of the other five. When MiAF was not the best, either MiRKAT or OMiAT was always among the top, where the power of OMiAT was predominantly driven by MiRKAT. When the sum of estimated mean absolute abundance of the associated OTUs was relatively small (around lower 60% among the 22 clusters), OMiAT had overall the best performance. In most cases, MiAF outperformed the inferior methods by a large margin even if it was not the best.

**FIGURE 3:**
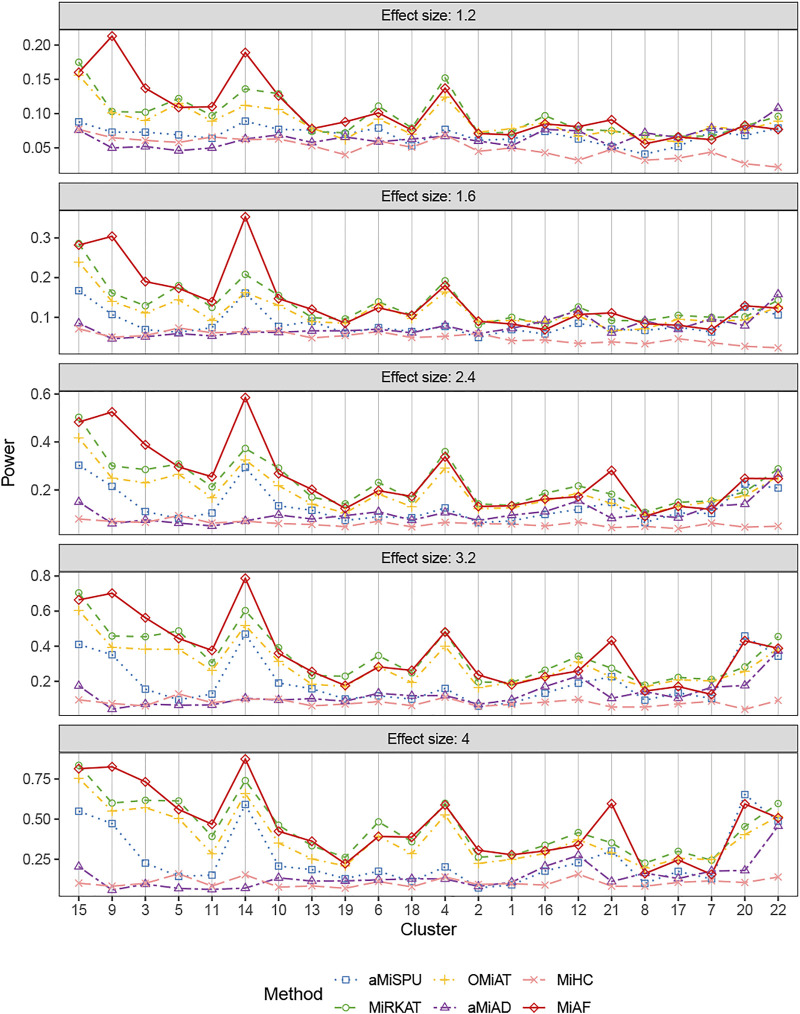
Power comparison for binary outcomes under the correlated case of scenario 1. A total of 616 OTUs were divided into 22 clusters. The covariates *Z*_*i*2_ and OTUs ***X***_***i⋅***_ were correlated. The effect size was set as 1.2, 1.6, 2.4, 3.2 and 4. The 22 clusters were sorted by the sum of estimated mean absolute abundance of the OTUs within the cluster that was truly associated from the greatest to the least.

The results for binary outcomes under scenario 1 with covariate *Z*_*i*2_ correlated with the OTU abundance were shown in Figure 3. Similar to the independent covariate case, the weighted tests possessed relatively higher statistical power with exceptions of clusters 7, 12, 16, 20, 21 and 22. In terms of the combined test, the advantage of MiAF over the other methods was more prominent than that in the independent case. In all the clusters except for cluster 7 and 8 where several methods were on par, MiAF achieved a dominant position over the other five methods or was a close second. We observed distinct advantage of MiAF in clusters 3, 9, 14 and 21, where MiAF had moderate power even when the effect size was small. It was interesting to see that the unweighted tests achieved their greatest power in cluster 22 where the mean OTU abundance was the lowest among all clusters. It confirmed that the unweighted tests are more powerful when clinical outcomes are associated with rare microbial taxa (Chen et al., 2012).

The simulation results for binary outcomes under scenario 2 and scenario 3 showed similar results, when the OTUs were partitioned into 10 or 29 clusters respectively. The power comparisons were shown in Supplementary Figures S2–S5. Our method, MiAF, achieved a dominant position over other methods consistently in correlated cases, where the existence of correlation between microbes and covariates is more biologically relevant in practice. MiAF performed equivalently well with OMiAT and MiRKAT in the independent cases when the sum of absolute abundances of the associated OTUs was relatively large (around top 70% and 30% among the 10 and 29 clusters respectively); while when the sum of absolute abundance of the associated OTUs was relatively small around lower 30% and 70% among the 10 and 29 clusters respectively, OMiAT was always in the lead. The power of MiAF was affected by the effect size and underlying association patterns, but not the direction of the effect (see the power comparison under the independent case of 10 clusters with positive and negative effects in Supplementary Figure S6).

**FIGURE 4:**
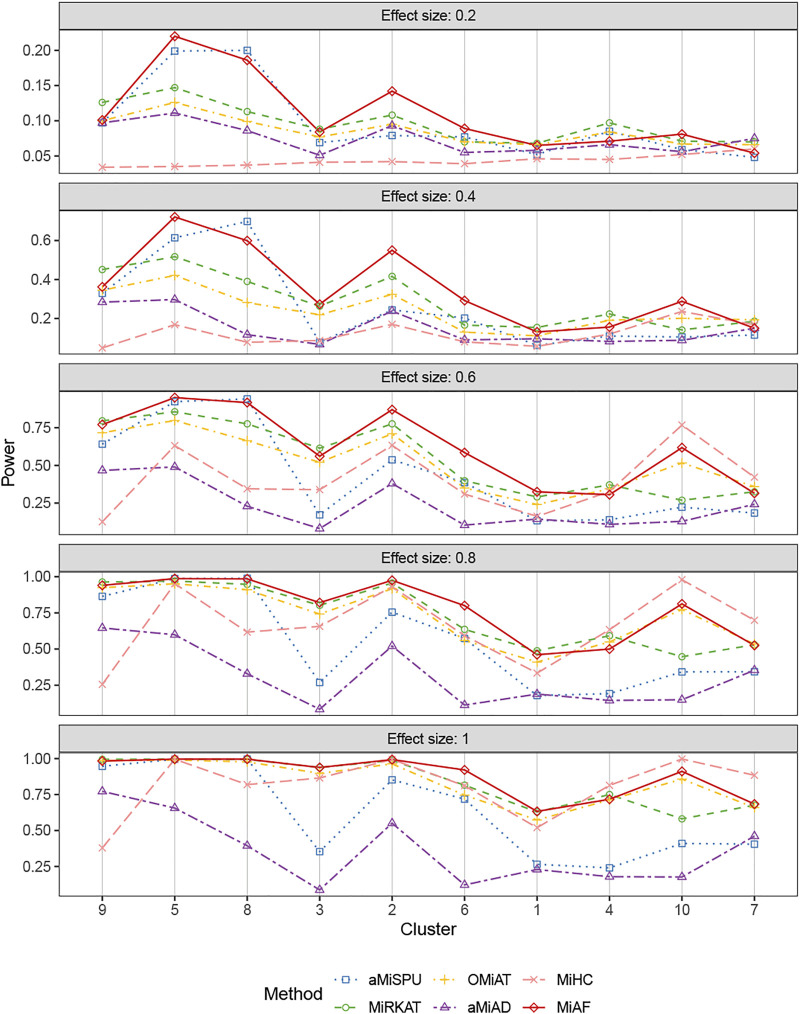
Power comparison for continuous outcomes under the independent case of scenario 2. A total of 616 OTUs were divided into 10 clusters. The covariates *Z*_*i*2_ and OTUs ***X***_***i⋅***_ were independent. The effect size was set as 0.2, 0.4, 0.6, 0.8 and 1. The 10 clusters were sorted by the sum of estimated mean absolute abundance of the OTUs within the cluster that was truly associated from the greatest to the least.

**FIGURE 5:**
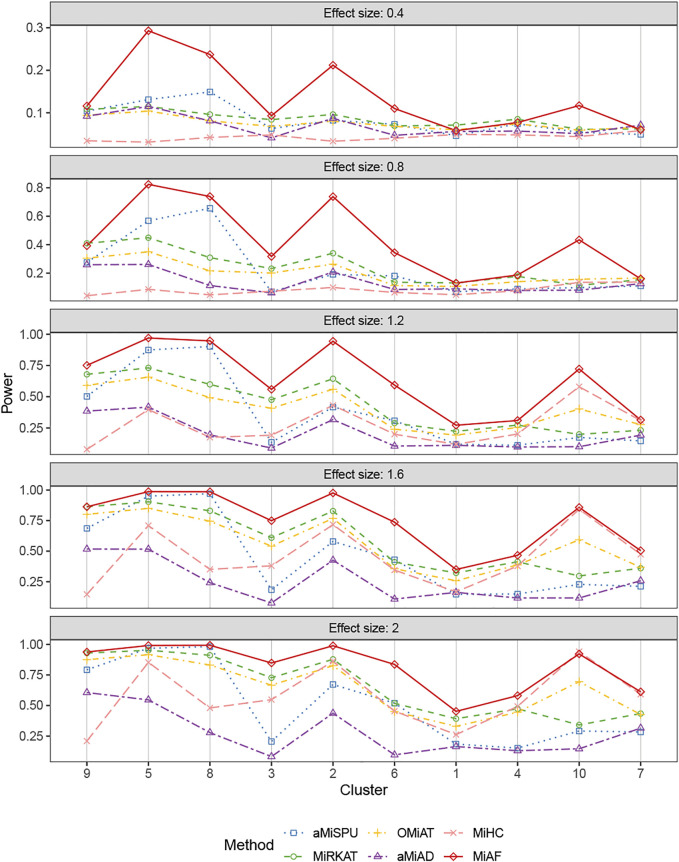
Power comparison for continuous outcomes under the correlated case of scenario 2. A total of 616 OTUs were divided into 10 clusters. The covariates *Z*_*i*2_ and OTUs ***X***_***i⋅***_ were correlated. The effect size was set as 0.4, 0.8, 1.2, 1.6 and 2. The 10 clusters were sorted by the sum of estimated mean absolute abundance of the OTUs within the cluster that was truly associated from the greatest to the least.

Figures 4, 5 displays the statistical power for continuous outcomes under scenario 2 for independent and correlated cases respectively, where 616 OTUs were divided into 10 clusters. The largest cluster consists of 171 OTUs (27.76%), and the sizes of the rest clusters are between 23 (3.41%) and 68 (11.04%) (see details in Supplementary Table S1A). In contrast to clearly different power comparison trend between independent and correlated cases for binary outcomes, the comparative power among the six methods was mainly affected by the associated clusters for continuous outcomes. MiAF continued to thrive when the sum of estimated mean absolute abundance of the OTUs within the selected cluster was relatively large (top 70% among 10 clusters for independent case and all 10 clusters for correlated case). We observed a great disparity in the performance of MiHC between binary and continuous outcomes, where MiHC was more capable of detecting the association between microbial communities and a continuous outcome. Besides, MiHC was barely able to detect the association for small effect size scenarios, and its power surged when the effect size raised to high level. MiHC had the greatest power among all the methods for relatively small sum of absolute abundance of the associated OTUs (around lower 2/3 and 1/2 for independent and correlated case respectively among 22 and 29 clusters), especially in some results with 22 or 29 clusters where the associated clusters tended to be in small size due to the large number of clustering (see Supplementary Figures S7–S10).

Empirical Type I error rates of the six methods across different simulation scenarios are shown in Table 2. Under the null model of independent case where the selected OTU cluster did not play a role, we had one unified assessment of type I error. For the correlated case, we averaged the type I error rates over all clusters within each scenario. The details of type I error rates for each cluster are provided in Supplementary Tables S2–S7 for binary and continuous responses respectively. Further, we investigated the Type I error rates for the independent case with QQ-plot of *p*-values in − log_10_ scale against a uniform distribution between 0 and 1 shown in Supplementary Figures S11, S12. We can see that the error rate was conservative for MiHC under binary responses, and that it was well under control for other methods (∼0.05) in general, which confirmed that our method is statistically valid.

**TABLE 2:** Type I error rates under independent case and mean type I error rates under correlated cases for both binary and continuous outcomes.

Simulation scenarios		aMiSPU	MiRKAT	OMiAT	aMiAD	MiHC	MiAF
Binary response	Independent case	0.059	0.048	0.047	0.050	0.025	0.055
	Correlated case, 10 clusters	0.046	0.042	0.046	0.043	0.032	0.051
	Correlated case, 22 clusters	0.048	0.047	0.049	0.047	0.032	0.048
	Correlated case, 29 clusters	0.050	0.046	0.047	0.048	0.025	0.048
Continuous response	Independent case	0.049	0.047	0.059	0.055	0.038	0.050
	Correlated case, 10 clusters	0.043	0.045	0.044	0.045	0.047	0.042
	Correlated case, 22 clusters	0.047	0.048	0.047	0.046	0.039	0.049
	Correlated case, 29 clusters	0.050	0.045	0.049	0.048	0.032	0.047

We compared the taxon selection results with the truth in our simulation settings. To demonstrate the performance of our heuristic taxon selection procedure, we took cluster 1 out of 10 clusters for continuous outcomes under independent case with effect size 1 as an example shown in Supplementary Figure S13 (see more results in Supplementary Figures S14–S16). The most often selected taxa over 1,000 replicates tended to be in high abundance, belonging to the truly associated cluster. MiAF had more difficulties in identifying associated taxa with low abundance, since the selection of low abundance taxa suffered from random noise, which renders the selection results of low abundance taxa unstable and unreliable. Therefore, the taxon selection result was more useful for abundant taxa, leading to more trustworthy insight into selecting taxa at relatively higher level of the phylogenetic tree in general, as their counts were aggregated from their descendants. To help navigate the taxa selection result and focus on abundant taxa only, we provide a visualization tool in our R package where the transparency of each branch was set according to the abundance of its node. The tendency to discover abundant associated taxa was consistent with the prominent performance of MiAF when the sum of absolute abundance of associated OTUs was large in the previous power results. We called the 10% most often selected taxa over 1,000 replicates as selected taxa in a simulation scenario, or otherwise as non-selected taxa to err on the conservative side. Under the independent case for continuous outcomes with effect size 1 where 616 OTUs were divided into 10 clusters, we also provided the sensitivity and specificity for abundant taxa, specifically taxa with abundance over 75%, 80% and 85% quantiles respectively in Supplementary Table S8. The overall specificity was considerably high, although the sensitivity was lower. It suggests that a subset of the associated taxa can be identified, and that we are unlikely to select wrong taxa based on our heuristic taxon selection algorithm. It should be noted that the taxa selection result is only exploratory and should not be over-interpreted.

### 3.2 Real Data Analysis

#### 3.2.1 Application to a Throat Microbiome Dataset

In our first real data application to demonstrate the utility of our proposed MiAF, we applied it and the competing methods to a profiling study of microbial communities in the upper respiratory tract to explore the effect of cigarette smoking (Charlson et al., 2010). In the study, microbiota were collected from the right and left nasopharynx and oropharynx of 29 smokers and 33 healthy non-smokers. After PCR amplification and QIIME pipeline, OTUs were constructed at 97% similarity. The preprocessed dataset is included in many statistical software packages such as GUniFrac (Chen et al., 2012), MiRKAT (Zhao et al., 2015) and MiSPU (Pan et al., 2014) as the testing data, which contain information on 856 OTUs in 60 samples (28 smokers and 32 nonsmokers), a slightly reduced data set from the original study. Our application used this dataset following the papers of MiRKAT and aMiSPU.

We applied MiRKAT, aMiSPU, OMiAT, aMiAD, MiHC and MiAF on this dataset to test the association between smoking and microbial community composition while controlling for gender. Table 3 presents *p*-values of these six methods. The combined MiAF generated a *p*-value of 0.0025, which confirmed the results published in previous studies that the association between the microbial community and smoking status remained significant while adjusting for possible confounders (Brook and Gober, 2008; Charlson et al., 2010; Schenck et al., 2016). MiHC was the only method that failed to detect such association among the six methods. The unweighted test of aMiSPU and MiAF_*u*_, as well as aMiAD using alpha diversity metrics Richness, Shannon, Simpson and phylogenetic diversity, alone failed to detect such association at significance level 0.05, although their corresponding combined results were significant. All the component tests of MiHC failed to detect any association in this dataset (see results of all the component tests of the six methods in Supplementary Table S9).

**TABLE 3:** P-values of aMiSPU, MiRKAT, OMiAT, aMiAD, MiHC and MiAF for the association test between smoking status and throat microbial community.

	aMiSPU	MiRKAT	OMiAT	aMiAD	MiHC	MiAF
p-value	0.0025	0.0046	0.0096	0.0167	0.2249	0.0025

Besides an overall evaluation of association, selecting associated taxa in a microbial community is also of interest. MiAF provides a heuristic taxon selection by choosing the top *h* taxa in the *p*-value combination step, where *h* is the minimizer of Equation 7. Supplementary Figure S17 shows the selected associated taxa for this throat microbiome dataset. The phylogenetic tree was plotted using the R package ggtree (Yu et al., 2018). MiAF detected 1 associated node to be under-presented in the smokers based on lower-tail *p*-values, and it detected 128 associated nodes to be over-presented from upper-tail *p*-values as well.

#### 3.2.2 Application to a Stool Microbiome Dataset

HIV infection induces substantial gut microbiome alterations. Lozupone et al. (Lozupone et al., 2013) revealed that HIV infection was associated with highly characteristic gut microbial community changes through 16 S rRNA sequencing of feces. In our second real data application, we downloaded the processed OTU data consisting of 10104 100% OTUs, i.e., ASVs, from the MicrobiomeHD database (Duvallet et al., 2017). After matching the samples to their clinical data, our analysis was conducted based on 22 HIV-infected individuals and 13 HIV-negative controls. After excluding OTUs with all zero counts in the 35 samples, 9,460 OTUs remained in the analysis. We built the phylogenetic tree using the QIIME2 pipeline (Bolyen et al., 2019).

We investigated the association between disease status and the overall microbial community composition using the six methods all based on 10,000 permutations, adjusting for potential confounder age. Table 4 shows the *p*-values generated by the six methods, where all the methods were able to detect the association at significance level 0.01 except for aMiSPU. While the unweighted test of aMiSPU and aMiAD using Shannon, Simpson and phylogenetic diversity, as well as the Simes test combined by MiHC failed to detect any association, the results of all the other component tests were significant at the 0.05 level (see details in Supplementary Table S10). As in the first application, we were also interested in finding individual taxa that are thought to be associated with HIV status. To this end, MiAF detected 224 and 57 associated nodes from under- and over-presented in the HIV-infected individuals respectively (phylogenetic tree plot was not included because it was hardly readable due to the large number of OTUs).

**TABLE 4:** P-values of aMiSPU, MiRKAT, OMiAT, aMiAD, MiHC and MiAF for the association test between HIV infectious status and gut microbial community.

	aMiSPU	MiRKAT	OMiAT	aMiAD	MiHC	MiAF
p-value	0.0114	0.0002	0.0001	0.0002	0.0001	0.0003

## 4 Discussion

In this paper, we proposed an adaptive *p*-value combination approach to construct a community-level association test from those that are OTU-level based. In general, combining OTU-level tests without adaptation or weighting may not generate comparable statistical power to sophisticated methods specifically designed for community-level association test. To demonstrate the usage and statistical power of the proposed approach, we constructed a community-level test, MiAF, by combining the *p*-values of univariate score tests using UniFrac-like and Bray-Curtis-like transformations and weighting scheme, and showed that its statistical power is comparable or better than methods specifically designed for community test. We chose to combine the *p*-values of score statistics to make it a fair comparison to the competing methods, because the performance of our method depends on the selection of univariate tests and the aMiSPU, MiRKAT and OMiAT test statistics can all be viewed as functions of the score statistics with similar weight selection.

It should be noted that the aMiSPU test can also be viewed as a test that combines OTU-level score test statistics. However, comparing to score statistic, *p*-value is a much more readily available statistic for various univariate testing methods. Although we demonstrated the usage of our proposed method using score tests, *p*-values are the quantities that we ultimately combine. This leads to the flexibility to our proposed framework since other tests can be combined into community-level tests, as long as they satisfy two conditions: 1) *p*-values (ideally one-sided) are available and can correctly control type I error; and 2) permutation or resampling methods exist to generate a reference distribution for the *p*-values while maintaining the correlation structure among the OTUs. We note that these two condition are met by a lot of tests, such as the tests in various regression models, where we can adopt a similar permutation procedure that permutes the residual of the condition of interest that regressed on the confounding covariates. For example, using this strategy, it will be relatively easy to construct a community-level test for survival outcome by combining any survival models, such as the Cox model or the accelerated failure time model. In addition, it is also possible to combine OTU-level tests to accommodate longitudinal outcomes or longitudinal microbiome measurements, which is our next topic in the future research.

A side product of our method is taxon selection, which is naturally provided by the minimizer. By plotting the selected taxa along the phylogenetic tree, we can see that they tend to occupy consecutive branches that leads to much fewer OTUs, which matches our intuition, because if a species is over-presented, the taxa in the upper hierarchy (such as genus, family, order, etc.) that contains the species should also be over-presented. However, this variable selection is only heuristic and is not the focus of this paper, because the *p*-values we combined are from univariate models, which perform marginal tests not conditional on other OTUs. Therefore, the OTUs selected are only marginally related to the outcome. It is still possible that some of the selected OTUs correlate to the outcome through other OTUs, which is a limitation of the proposed method. Another limitation is the relatively slow computational speed compared to aMiSPU, MiRKAT, OMiAT and MiHC when there are a large number of taxa, but it is faster than aMiAD. When analyzing the data set of our first real data application in a laptop with 8-core CPU and 8 GB unified memory, it takes 7 s for aMiSPU, 3 s for MiRKAT, 17 s for OMiAT, 21 s for MiHC, 5 min 45 s for MiAF, and 7 min 41 s for aMiAD. Despite relatively slower computational speed, it is still computationally feasible to apply MiAF to real data sets given that MiAF will only need to be performed once on the data set to test the association. Improving the computational speed of our method is one of our future work.

## Data Availability

Publicly available datasets were analyzed in this study. This data can be found here: The throat microbiome dataset is available in R package MiSPU, and the stool microbiome dataset (hiv_lozupone) is openly available in MicrobiomeHD at https://zenodo.org/record/1146764#.Ylh_1i1h1PM.
