# Supplementary material for: An Adaptive and Robust Test for Microbial Community Analysis
Source: Front Genet. 2022 May 19;13:846258. doi: 10.3389/fgene.2022.846258 (PMC9162041; doi:10.3389/fgene.2022.846258)
Supplement: Supplementary file 1 [file DataSheet1.pdf]

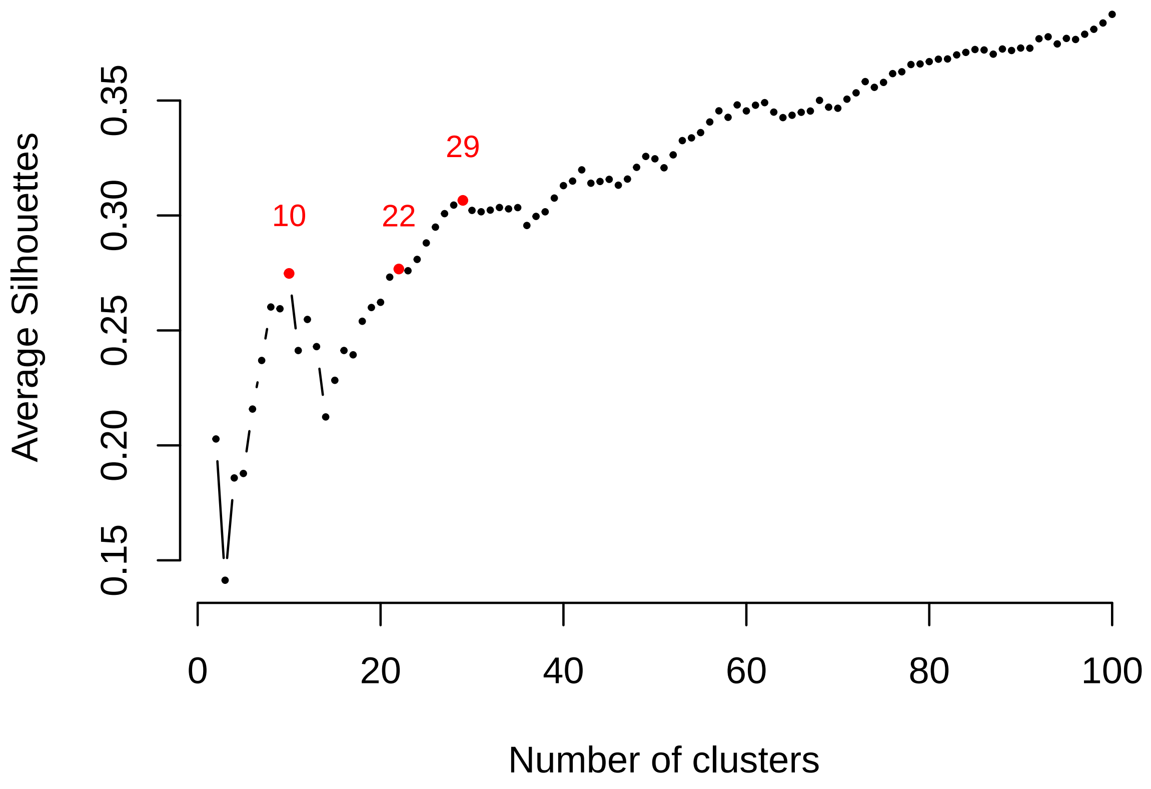

Figure S1: Mean Silhouette values for different numbers of clusters on 616 OTUs.

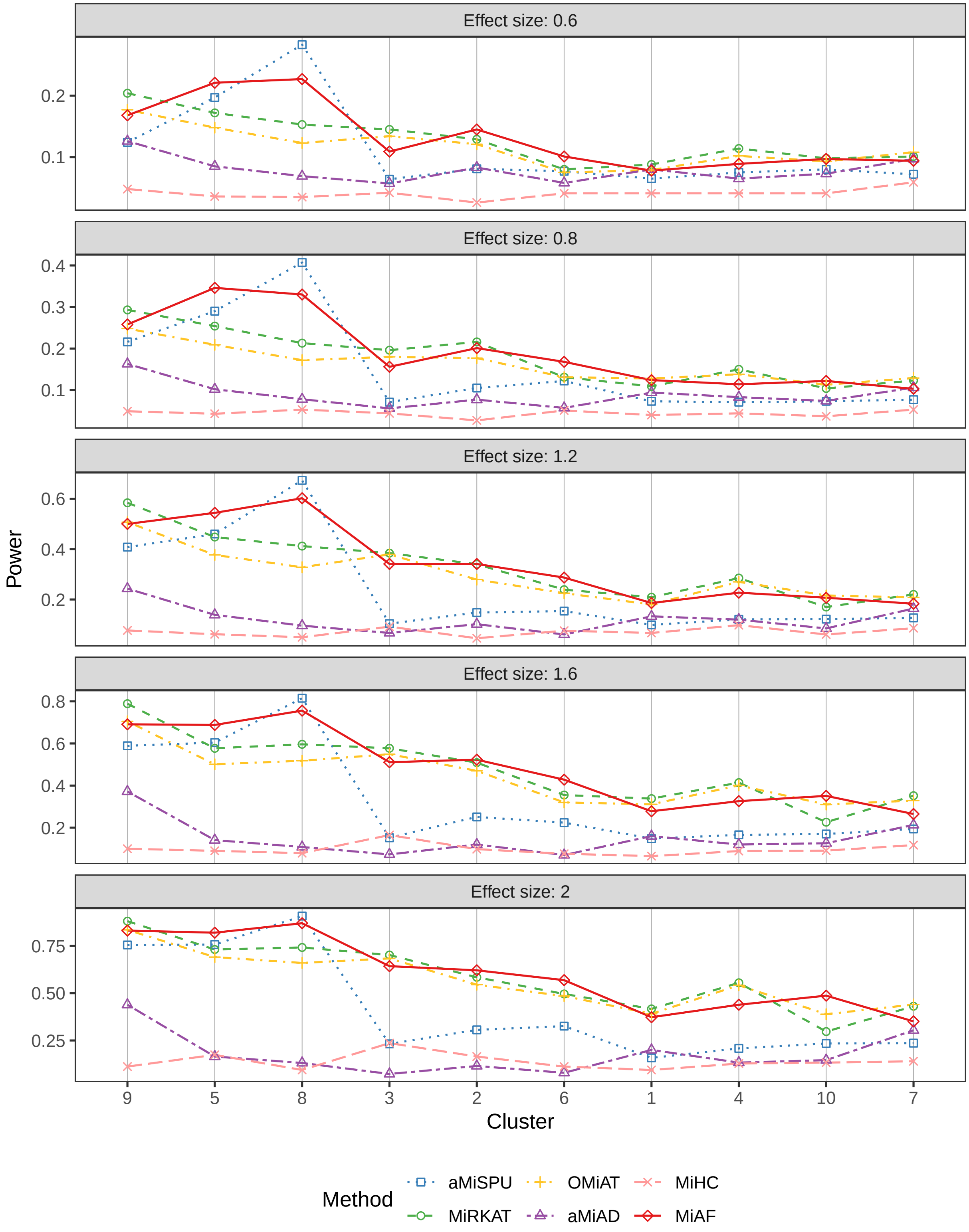

Figure S2: Power comparison for binary outcomes under the independent case of scenario 2. 616 OTUs were divided into 10 clusters. The covariates Z_{i2} and OTUs $\boldsymbol{X}_{i\cdot}$ were independent. The effect size was set as 0.6, 0.8, 1.2, 1.6 and 2. The 10 clusters were sorted by the sum of estimated mean absolute abundance of the OTUs within the cluster that was truly associated from the greatest to the least.

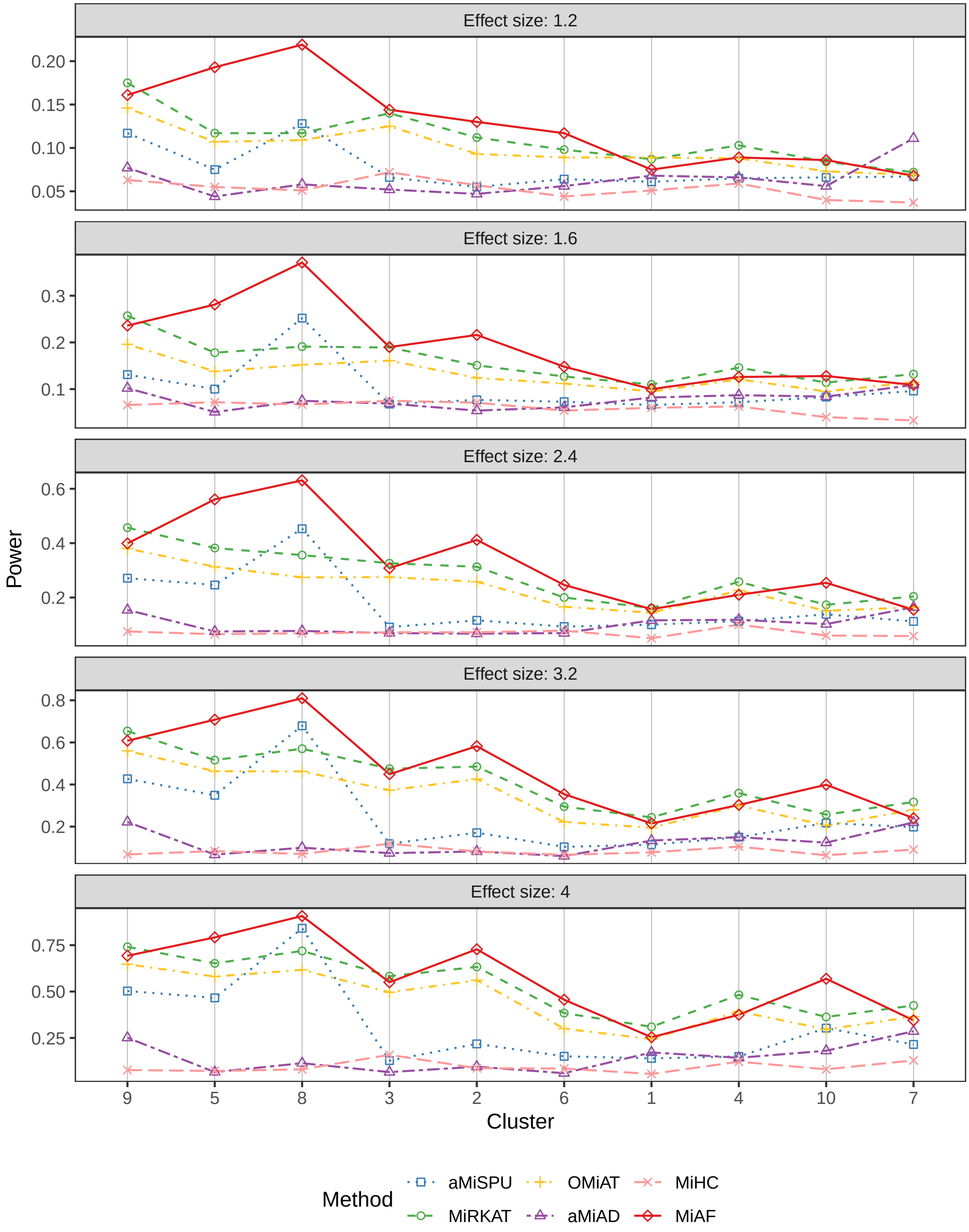

Figure S3: Power comparison for binary outcomes under the correlated case of scenario 2. 616 OTUs were divided into 10 clusters. The covariates Z_{i2} and OTUs $\boldsymbol{X}_{i\cdot}$ were correlated. The effect size was set as 1.2, 1.6, 2.4, 3.2 and 4. The 10 clusters were sorted by the sum of estimated mean absolute abundance of the OTUs within the cluster that was truly associated from the greatest to the least.

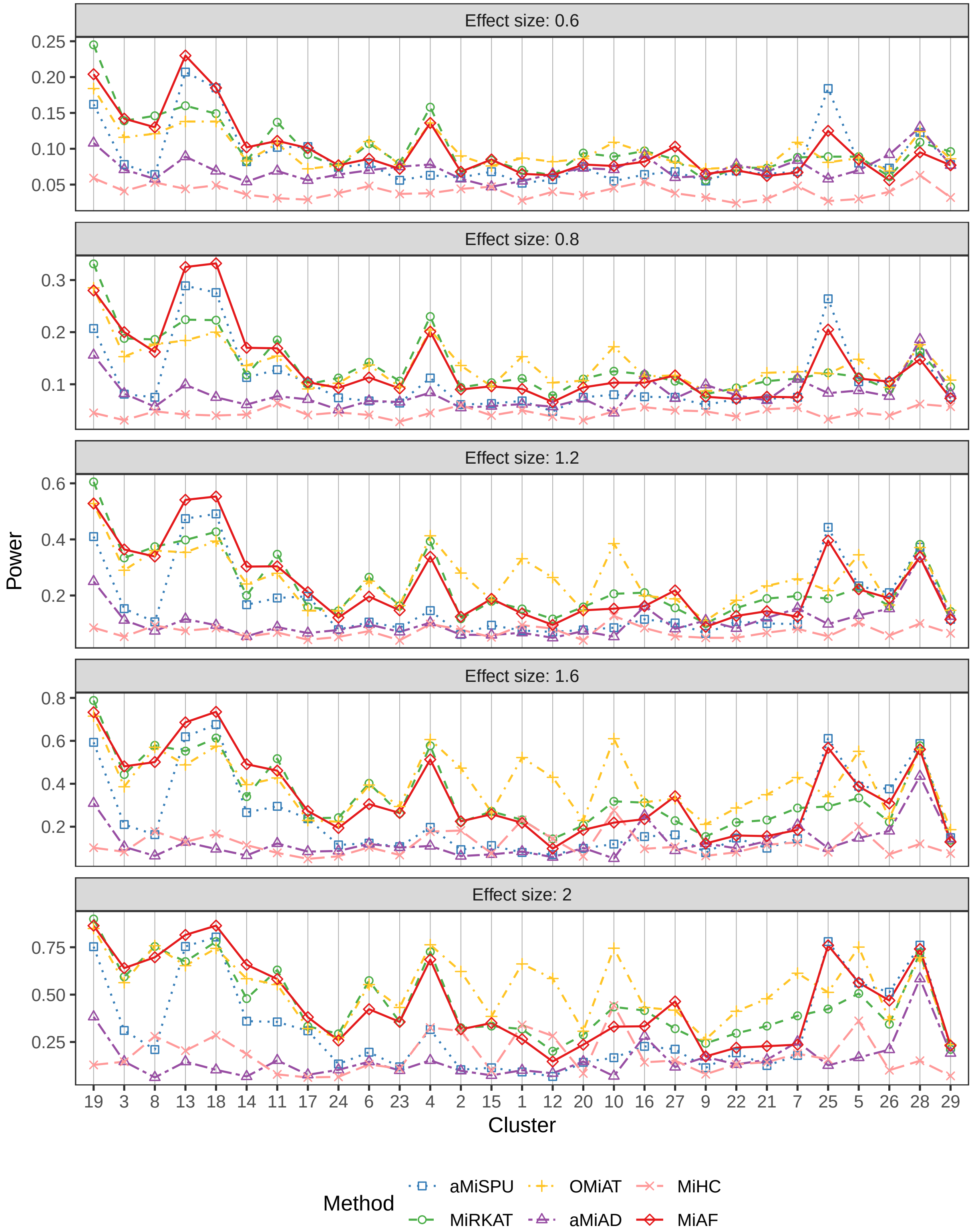

Figure S4: Power comparison for binary outcomes under the independent case of scenario 3. 616 OTUs were divided into 29 clusters. The covariates Z_{i2} and OTUs $\boldsymbol{X}_{i\cdot}$ were independent. The effect size was set as 0.6, 0.8, 1.2, 1.6 and 2. The 29 clusters were sorted by the sum of estimated mean absolute abundance of the OTUs within the cluster that was truly associated from the greatest to the least.

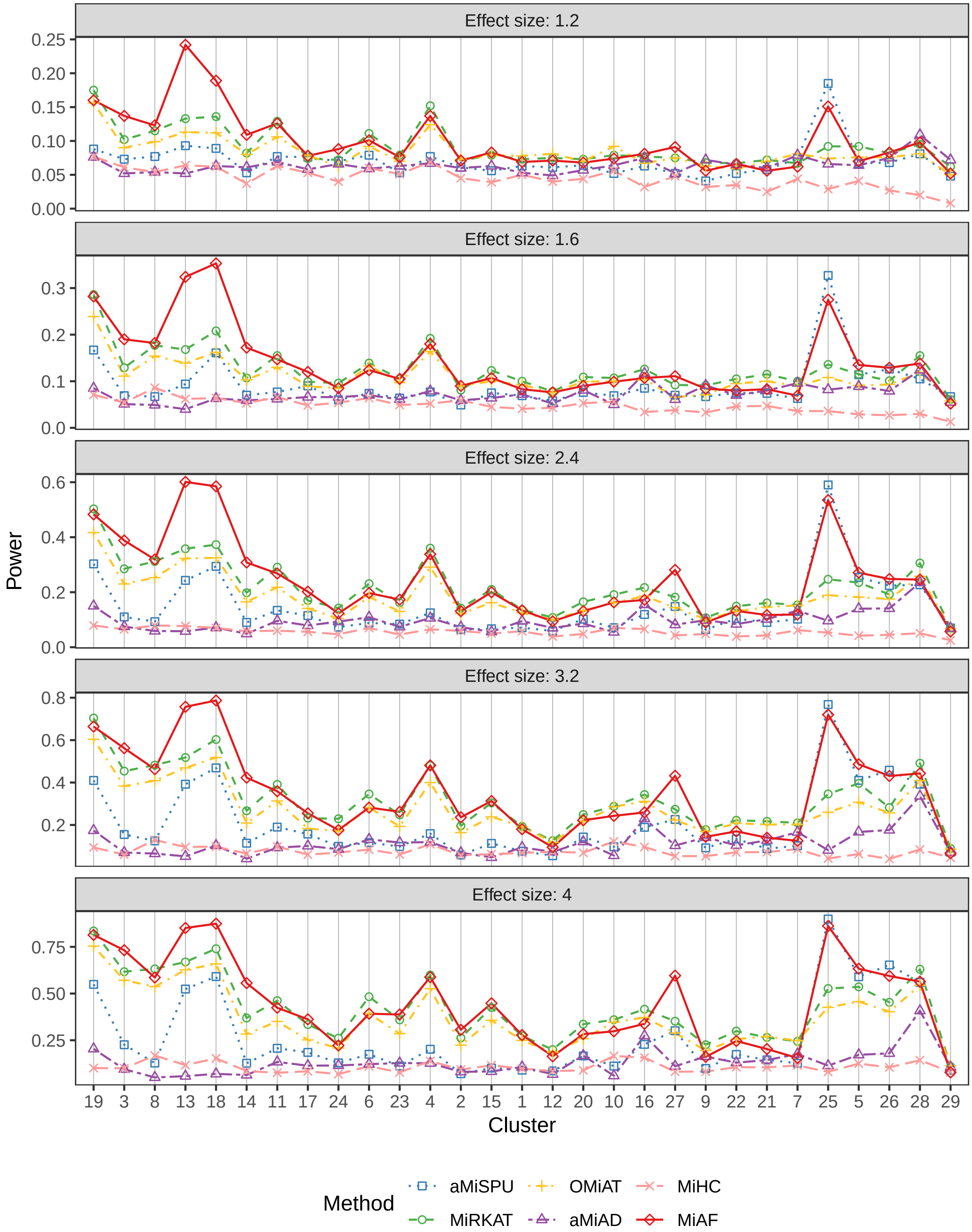

Figure S5: Power comparison for binary outcomes under the correlated case of scenario 3. 616 OTUs were divided into 29 clusters. The covariates Z_{i2} and OTUs $\boldsymbol{X}_{i\cdot}$ were correlated. The effect size was set as 1.2, 1.6, 2.4, 3.2 and 4. The 29 clusters were sorted by the sum of estimated mean absolute abundance of the OTUs within the cluster that was truly associated from the greatest to the least.

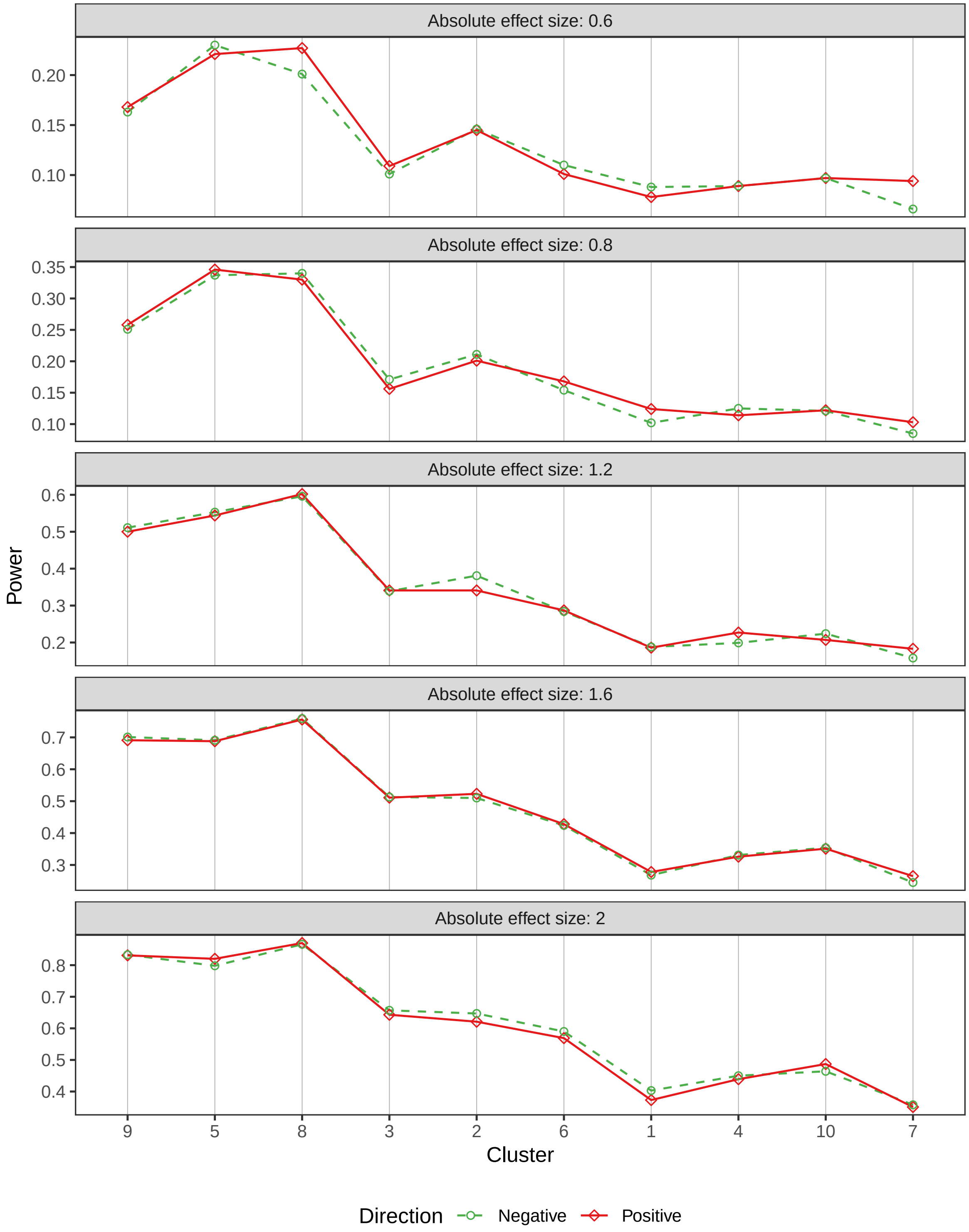

Figure S6: Power comparison for binary outcomes under the independent case of scenario 2 with both positive and negative effect directions.616 OTUs were divided into 10 clusters. The covariates Z_{i2} and OTUs $\boldsymbol{X}_{i\cdot}$ were independent. The effect size for positive direction was set as 0.6, 0.8, 1.2, 1.6 and 2, and the effect size for negative direction was set as -0.6, -0.8, -1.2, -1.6 and -2. The 10 clusters were sorted by the sum of estimated mean absolute abundance of the OTUs within the cluster that was truly associated from the greatest to the least.

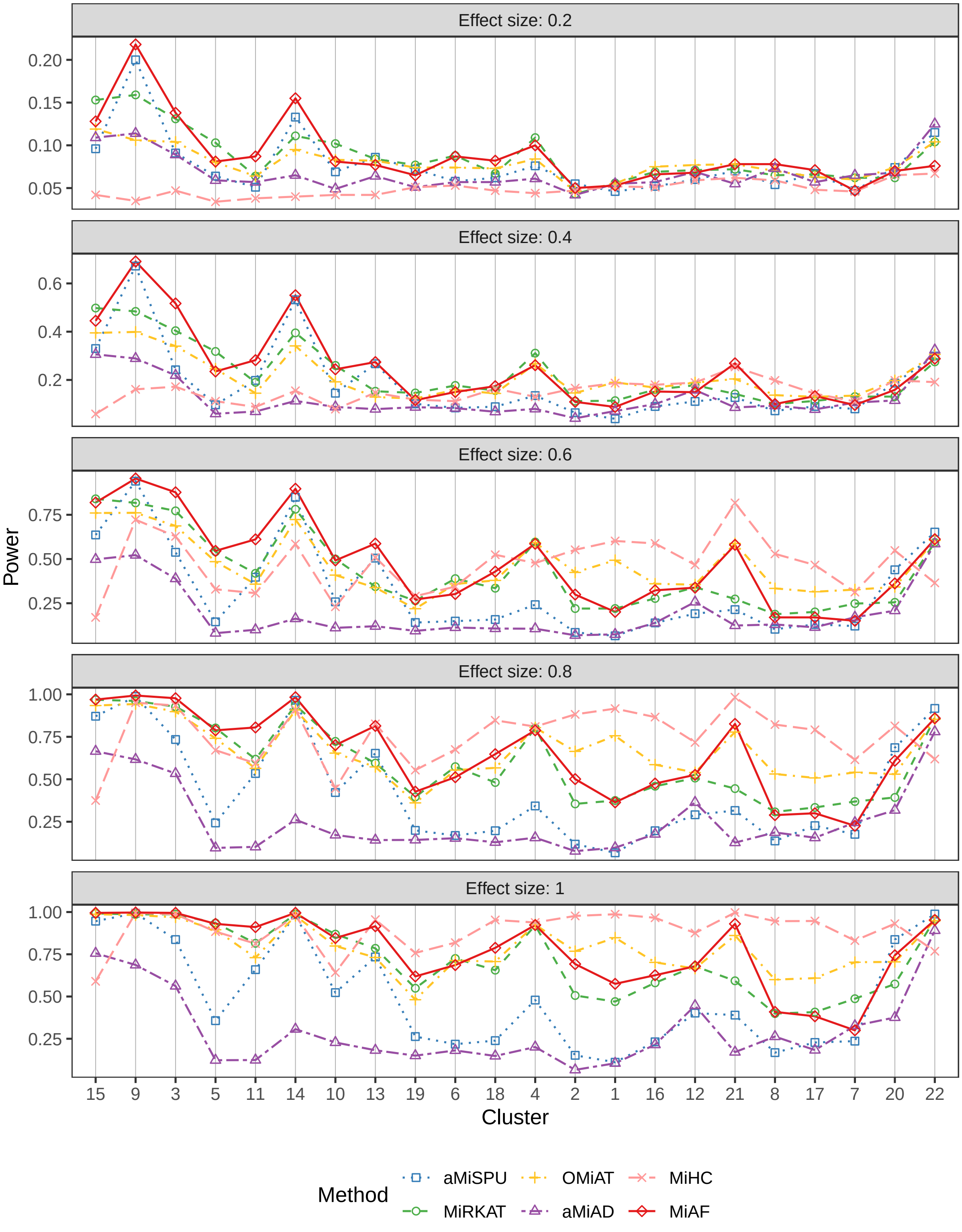

Figure S7: Power comparison for continuous outcomes under the independent case of scenario 1. 616 OTUs were divided into 22 clusters. The covariates Z_{i2} and OTUs $\boldsymbol{X}_{i\cdot}$ were independent. The effect size was set as 0.2, 0.4, 0.6, 0.8 and 1. The 22 clusters were sorted by the sum of estimated mean absolute abundance of the OTUs within the cluster that was truly associated from the greatest to the least.

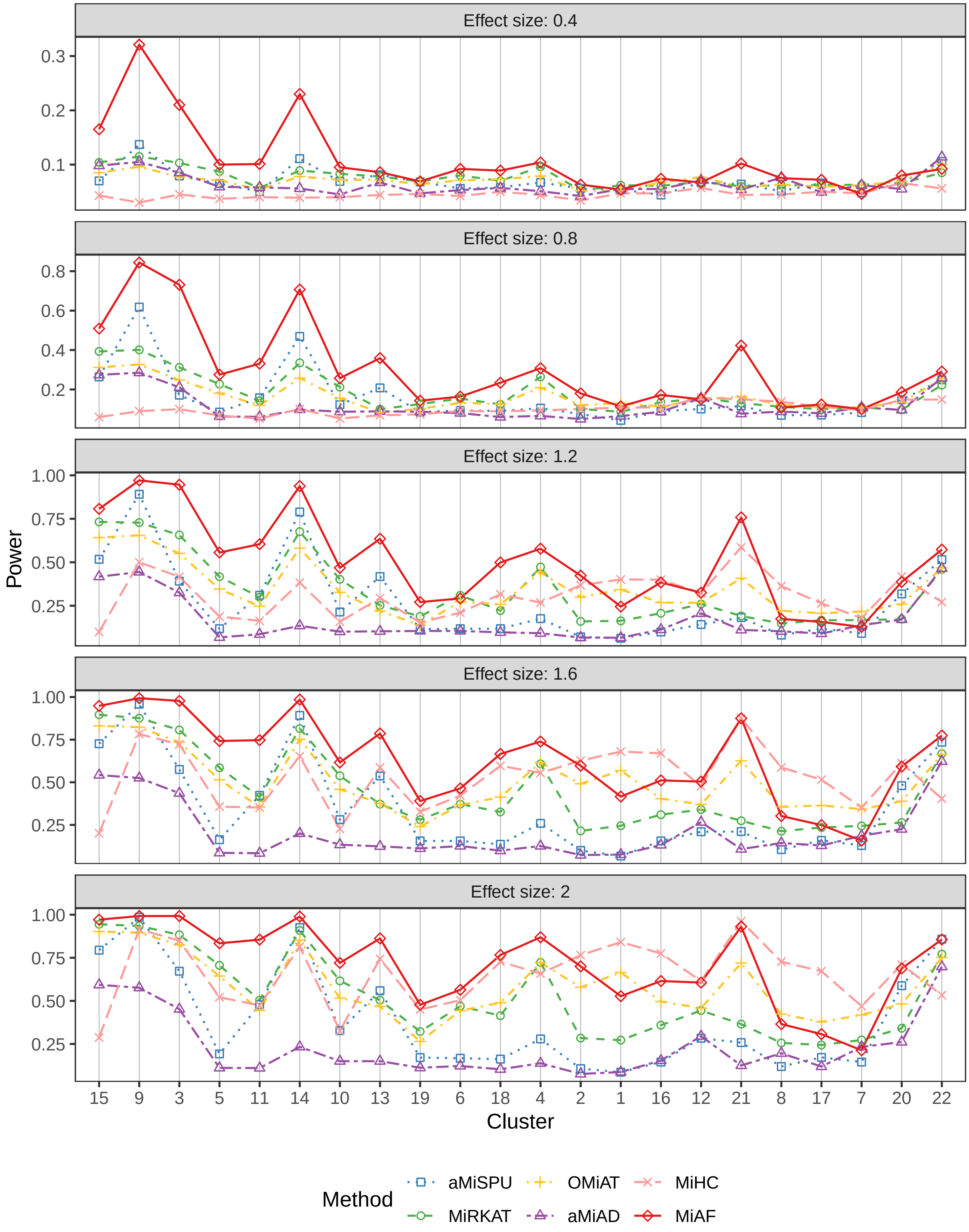

Figure S8: Power comparison for continuous outcomes under the correlated case of scenario 1. 616 OTUs were divided into 22 clusters. The covariates Z_{i2} and OTUs $\boldsymbol{X}_{i\cdot}$ were correlated. The effect size was set as 0.4, 0.8, 1.2, 1.6 and 2. The 22 clusters were sorted by the sum of estimated mean absolute abundance of the OTUs within the cluster that was truly associated from the greatest to the least.

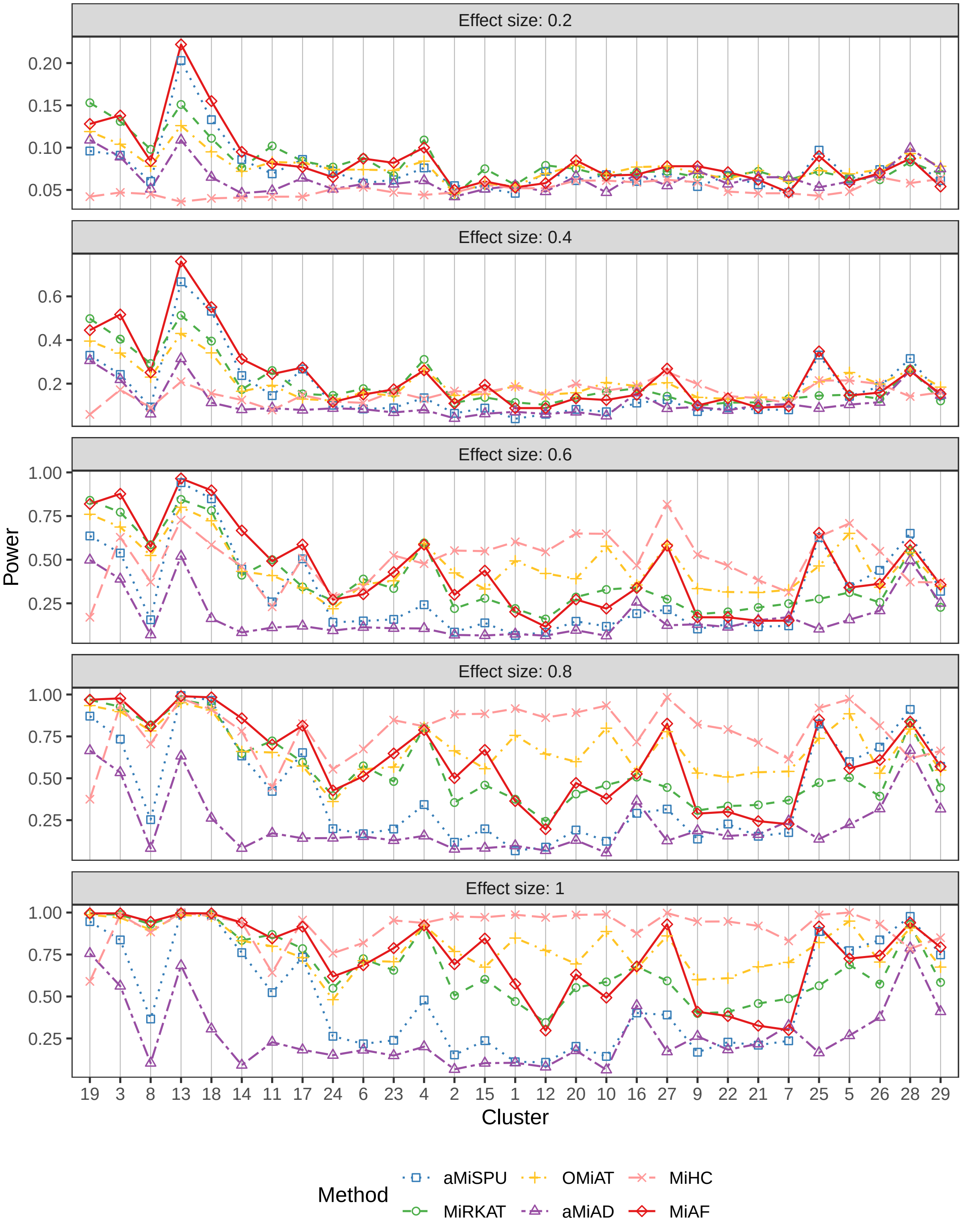

Figure S9: Power comparison for continuous outcomes under the independent case of scenario 3. 616 OTUs were divided into 29 clusters. The covariates Z_{i2} and OTUs $\boldsymbol{X}_{i\cdot}$ were independent. The effect size was set as 0.2, 0.4, 0.6, 0.8 and 1. The 29 clusters were sorted by the sum of estimated mean absolute abundance of the OTUs within the cluster that was truly associated from the greatest to the least.

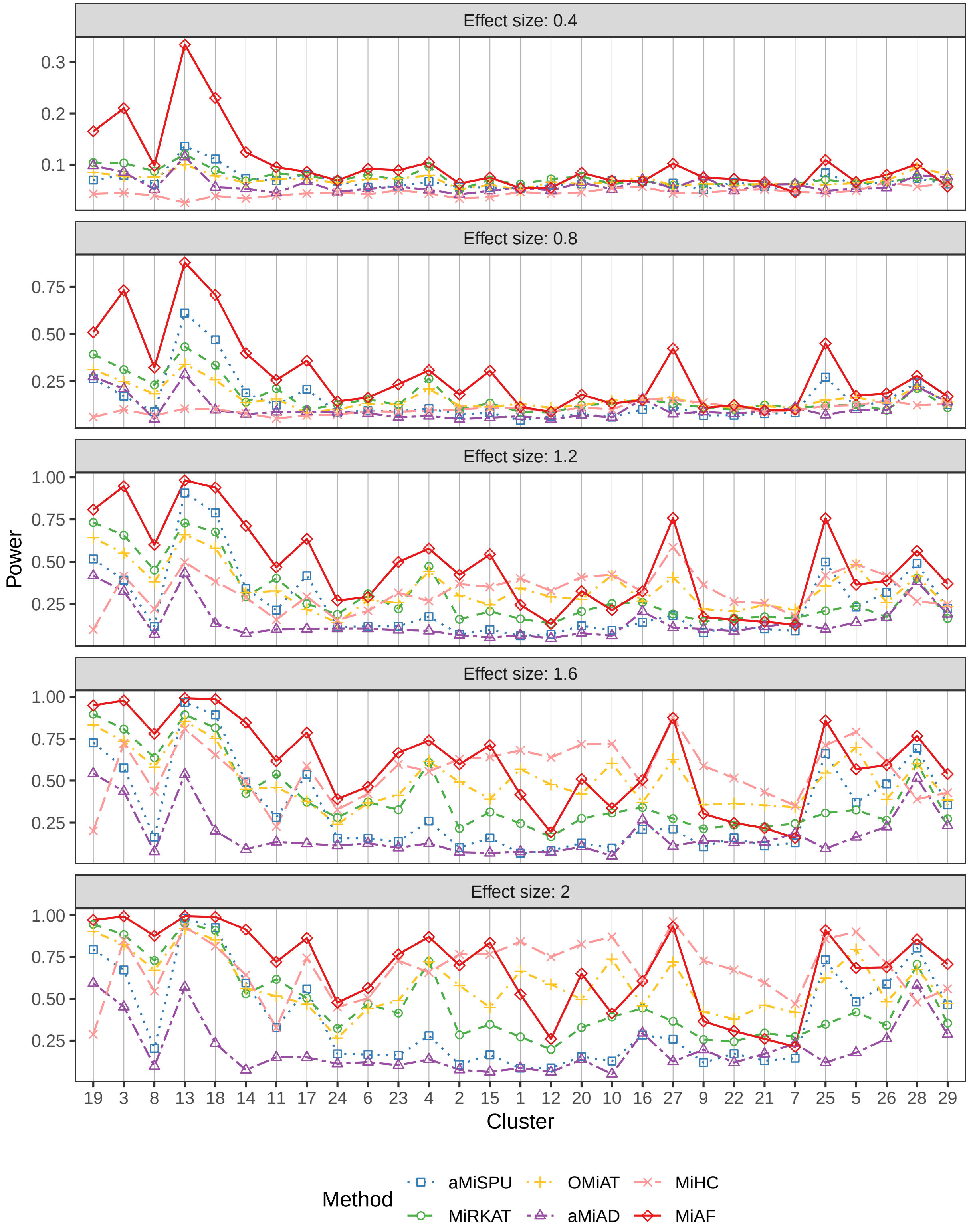

Figure S10: Power comparison for continuous outcomes under the correlated case of scenario 3. 616 OTUs were divided into 29 clusters. The covariates Z_{i2} and OTUs $\boldsymbol{X}_{i\cdot}$ were correlated. The effect size was set as 0.4, 0.8, 1.2, 1.6 and 2. The 29 clusters were sorted by the sum of estimated mean absolute abundance of the OTUs within the cluster that was truly associated from the greatest to the least.

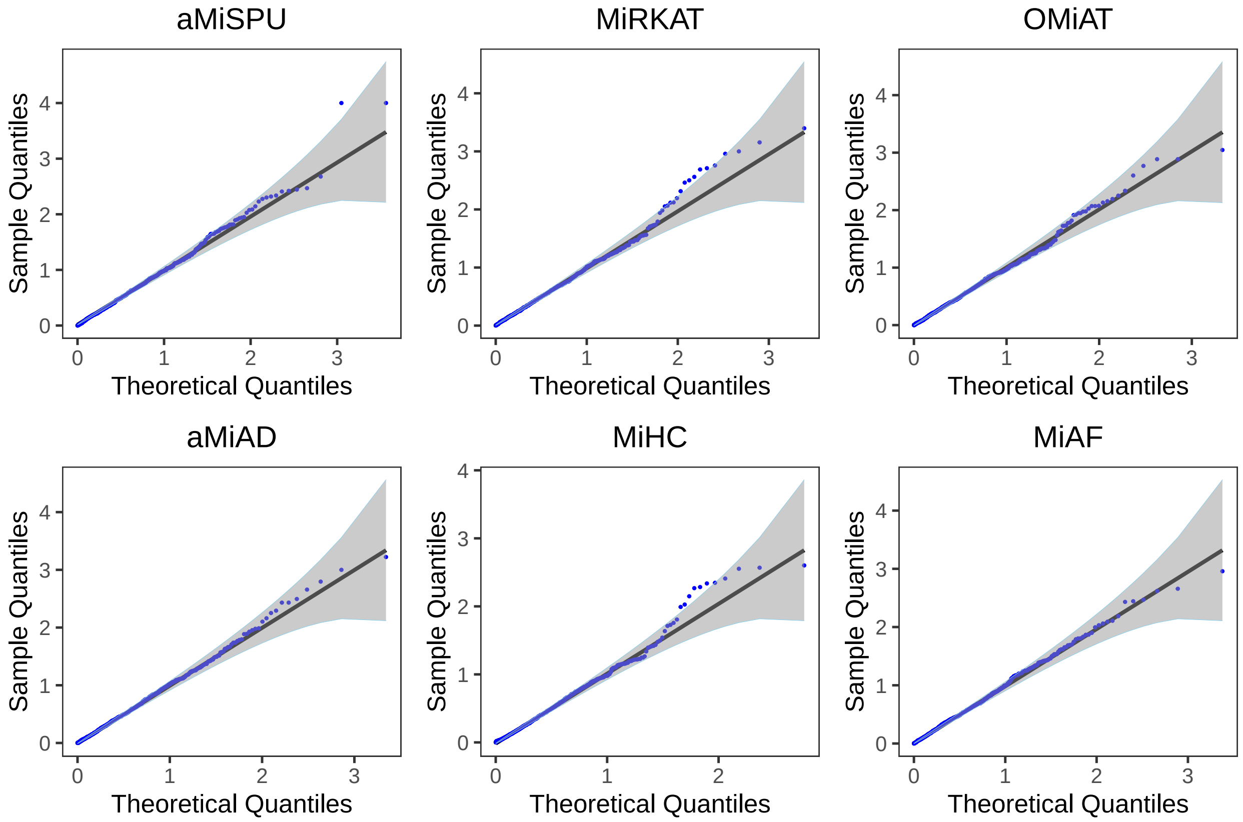

Figure S11: QQ-plot of the p-values in $-\log_{10}$ scale for the independent case under binary responses. The 95% pointwise confidence band in grey is plotted assuming that p-values follow a uniform distribution. The black line represents the reference line.

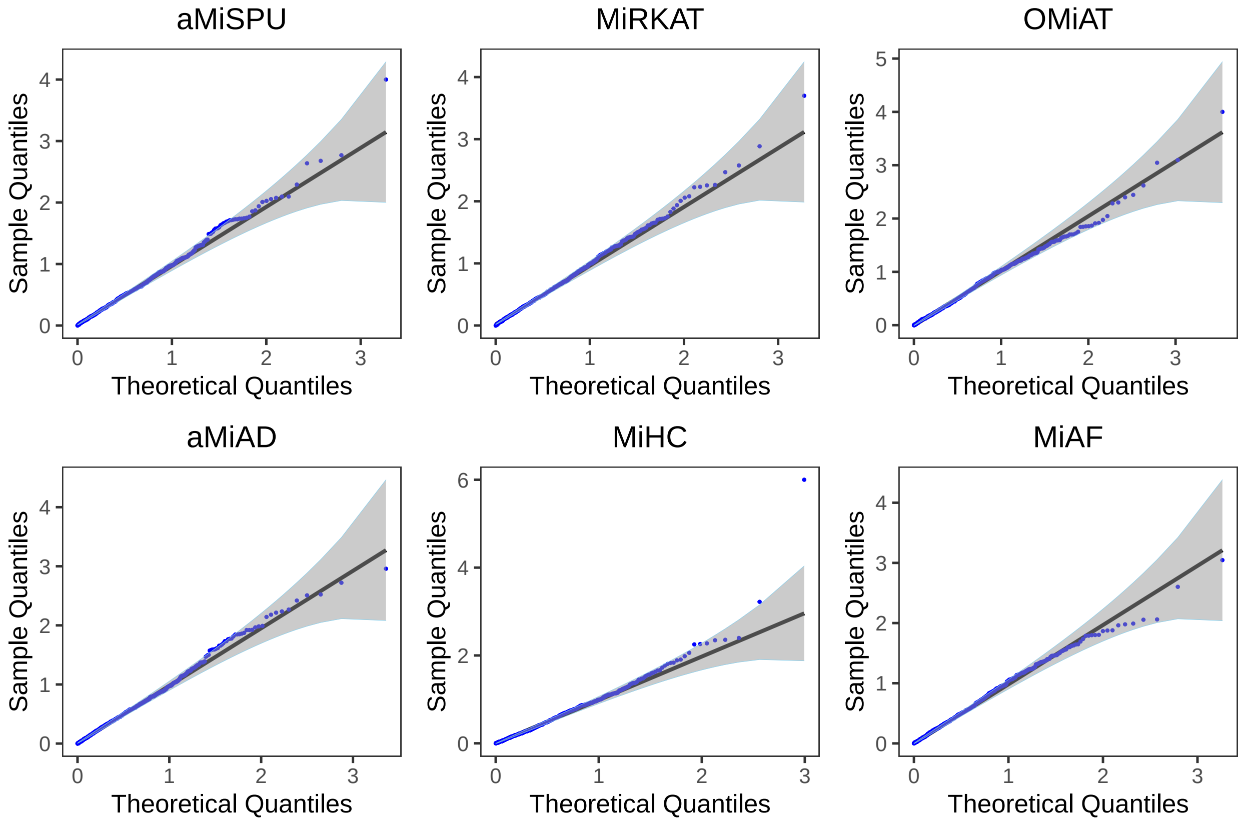

Figure S12: QQ-plot of the p-values in $-\log_{10}$ scale for the independent case under continuous responses. The 95% pointwise confidence band in grey is plotted assuming that p-values follow a uniform distribution. The black line represents the reference line.

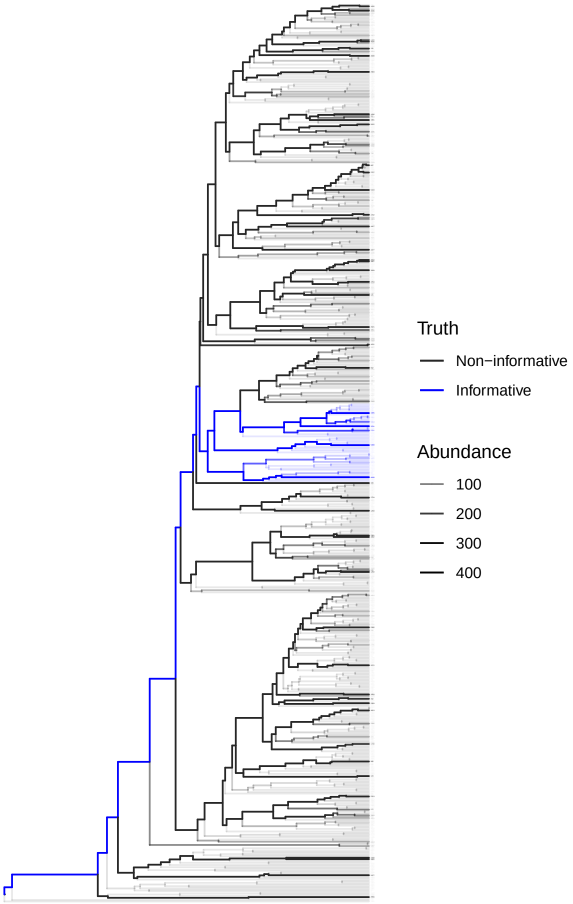

(a) True association signals.

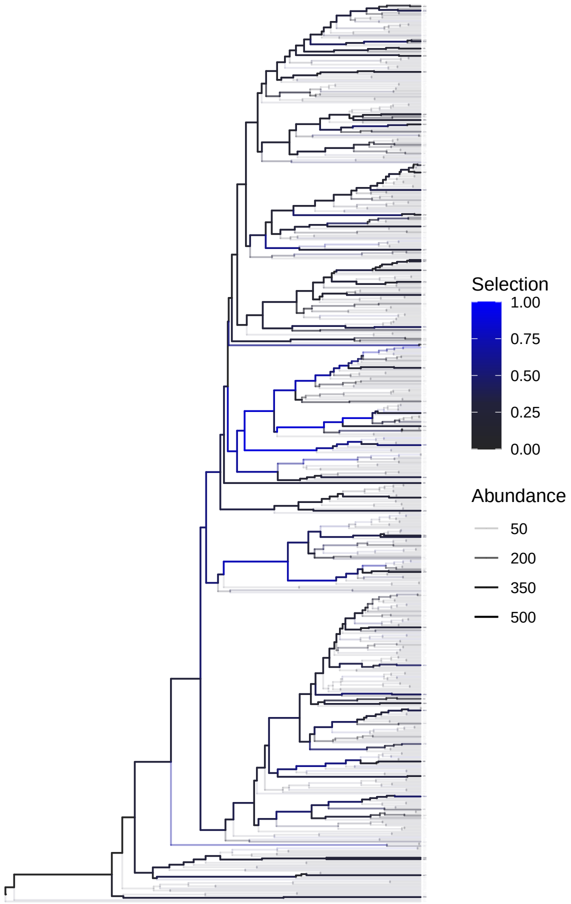

(b) Taxon selection results in simulation.

Figure S13: (a)True association signals of cluster 1 under scenario 2. 616 OTUs were divided into 10 clusters. Black edges represent non-associated signals, and blue edges represent associated signals in the simulation setting. **(b)Taxa selection results of cluster 1 for continuous outcomes under independent case of scenario 2 with effect size 1.** Black edges represent non-detected signals, and blue edges represent taxa selected as positive associated based on 1,000 replicates. The transparency of leaf nodes represent their abundance levels, with more sparse taxa being more transparent.

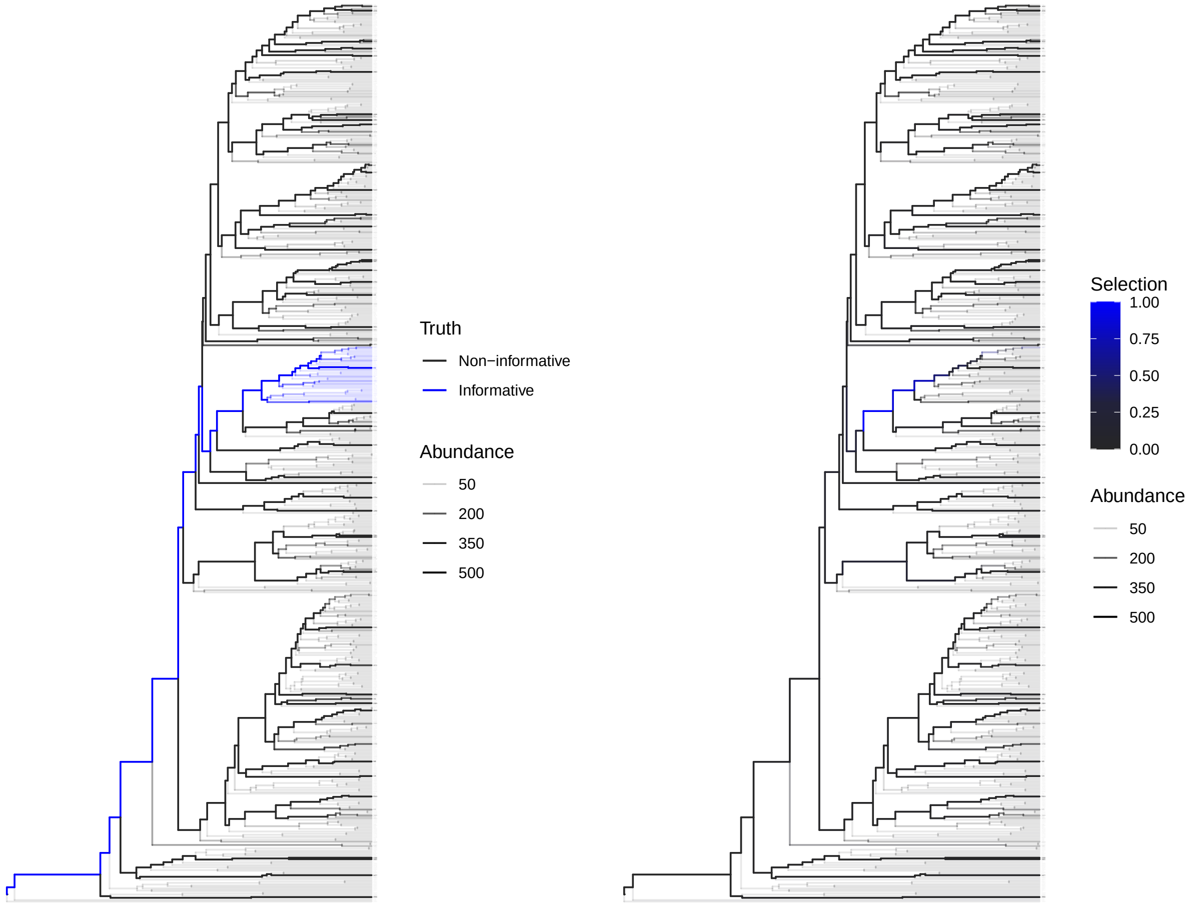

(a) True association signals.

(b) Taxon selection results in simulation.

Figure S14: (a)True association signals of cluster 2 under scenario 2. 616 OTUs were divided into 10 clusters. Black edges represent non-associated signals, and blue edges represent associated signals in the simulation setting. **(b)Taxa selection results of cluster 2 for continuous outcomes under independent case of scenario 2 with effect size 1.** Black edges represent non-detected signals, and blue edges represent taxa selected as positive associated based on 1,000 replicates. The transparency of leaf nodes represent their abundance levels, with more sparse taxa being more transparent.

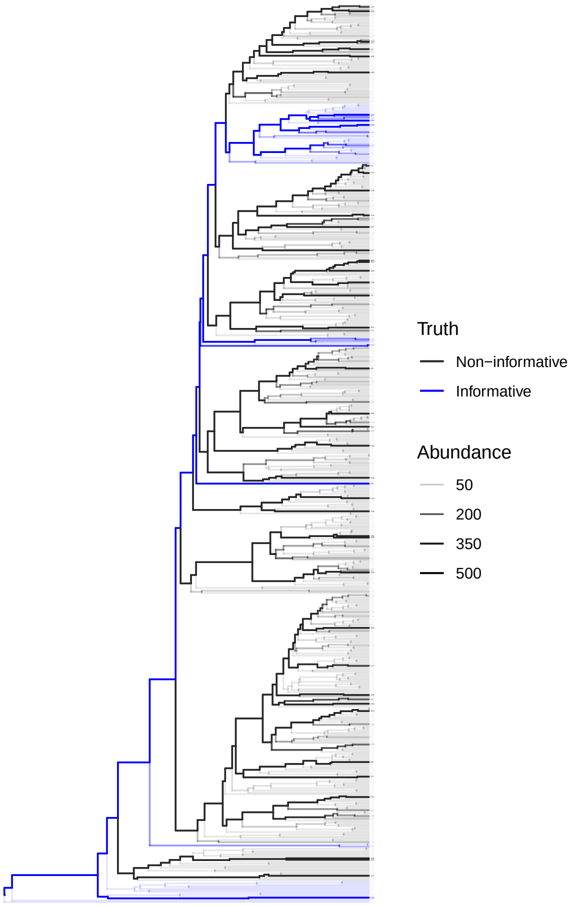

(a) True association signals.

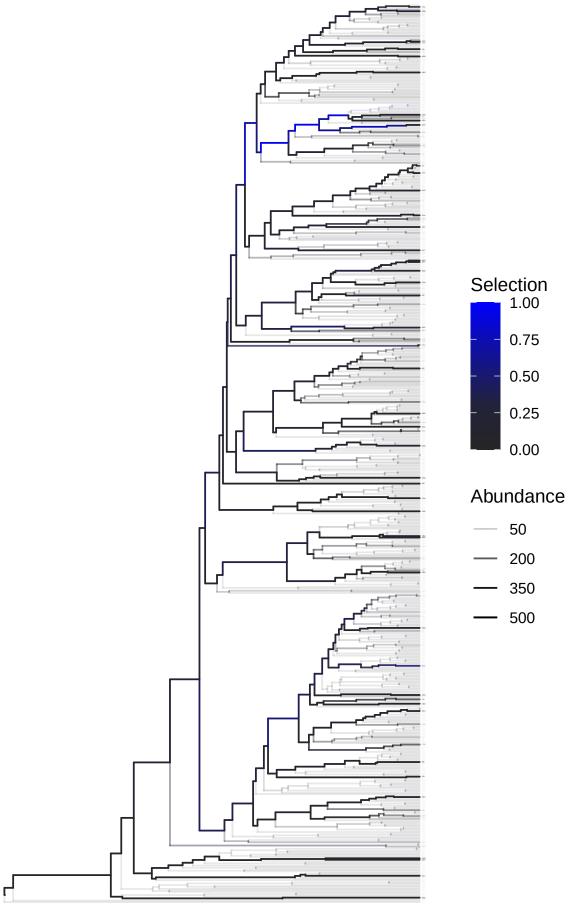

(b) Taxon selection results in simulation.

Figure S15: (a)True association signals of cluster 3 under scenario 2. 616 OTUs were divided into 10 clusters. Black edges represent non-associated signals, and blue edges represent associated signals in the simulation setting. Note that if we reorganize the tree, i.e., switch the position of left and right child, the taxa belonging to cluster 3 can be displayed as gathering together. **(b)Taxa selection results of cluster 3 for continuous outcomes under independent case of scenario 2 with effect size 1.** Black edges represent non-detected signals, and blue edges represent taxa selected as positive associated based on 1,000 replicates. The transparency of leaf nodes represent their abundance levels, with more sparse taxa being more transparent.

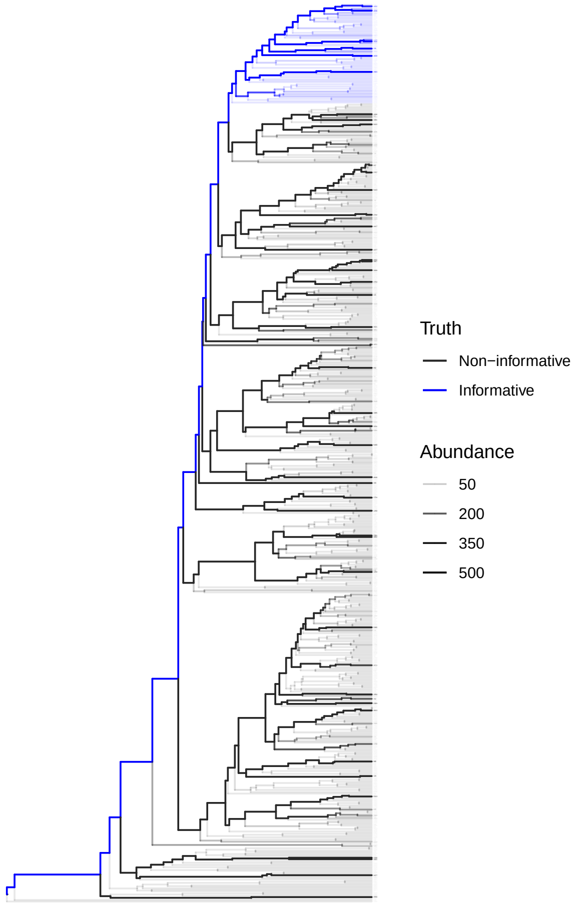

(a) True association signals.

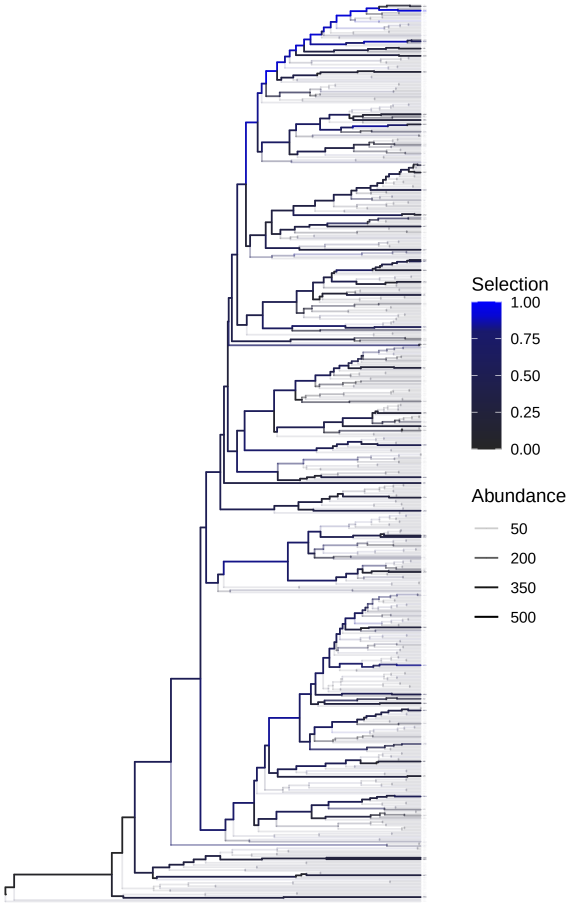

(b) Taxon selection results in simulation.

Figure S16: (a)True association signals of cluster 4 under scenario 2. 616 OTUs were divided into 10 clusters. Black edges represent non-associated signals, and blue edges represent associated signals in the simulation setting. **(b)Taxa selection results of cluster 4 for continuous outcomes under independent case of scenario 2 with effect size 1.** Black edges represent non-detected signals, and blue edges represent taxa selected as positive associated based on 1,000 replicates. The transparency of leaf nodes represent their abundance levels, with more sparse taxa being more transparent.

Table S1: Summary of cluster size (percentage), estimated mean absolute abundance and the sum of estimated mean absolute abundance within each cluster for 10, 22 and 29 clusters.

(a) 10 clusters.

Cluster	Cluster Size	Mean Abundance	Total Abundance
1	54 (8.8%)	1.04	56.3
2	39 (6.3%)	1.95	75.9
3	67 (10.9%)	1.20	80.2
4	68 (11.0%)	0.57	38.5
5	66 (10.7%)	2.02	133.0
6	53 (8.6%)	1.30	69.1
7	21 (3.4%)	0.25	5.2
8	54 (8.8%)	1.97	107.0
9	171 (27.8%)	1.78	305.0
10	23(3.7%)	0.23	5.2

(b) 22 clusters.

Cluster	Cluster Size	Mean Abundance	Total Abundance
1	20 (3.2%)	0.64	12.7
2	11 (1.8%)	1.82	20.0
3	39 (6.3%)	1.95	75.9
4	23 (3.7%)	1.02	23.5
5	38 (6.2%)	2.00	75.9
6	55 (8.9%)	0.67	36.8
7	13 (2.1%)	0.13	1.7
8	14 (2.3%)	0.29	4.0
9	29 (4.7%)	2.90	84.2
10	37 (6.0%)	1.32	48.9
11	53 (8.6%)	1.30	69.1
12	21 (3.4%)	0.25	5.2
13	36 (5.8%)	1.22	43.9
14	18 (2.9%)	3.48	62.6
15	86 (1.4%)	2.61	224.0
16	26 (4.2%)	0.42	11.0
17	10 (1.6%)	0.36	3.6
18	18 (2.9%)	1.37	24.7
19	31 (5.0%)	1.33	41.3
20	6 (1.0%)	0.08	0.5
21	17 (2.8%)	0.28	4.8
22	15 (2.4%)	0.02	0.3

(c) 29 clusters.

Cluster	Cluster Size	Mean Abundance	Total Abundance
1	20 (3.2%)	0.64	12.7
2	11 (1.8%)	1.82	20.0
3	39 (6.3%)	1.95	75.9
4	23 (3.7%)	1.02	23.5
5	5 (0.8%)	0.16	0.8
6	55 (8.9%)	0.67	36.8
7	13 (2.1%)	0.13	1.7
8	30 (4.9%)	2.45	73.5
9	14 (2.3%)	0.29	4.0
10	6 (1.0%)	0.93	5.6
11	37 (6.0%)	1.32	48.9
12	16 (2.6%)	0.68	11.0
13	7 (1.1%)	9.65	67.5
14	45 (7.3%)	1.19	53.4
15	8 (1.3%)	1.96	15.7
16	21 (3.4%)	0.25	5.17
17	36 (5.8%)	1.22	43.9
18	18 (2.9%)	3.48	62.6
19	86 (1.4%)	2.61	224.0
20	18 (2.9%)	0.48	8.7
21	8 (1.3%)	0.29	2.3
22	10 (1.6%)	0.36	3.56
23	18 (2.9%)	1.37	24.7
24	31 (5.0%)	1.33	41.3
25	3 (0.5%)	0.53	1.6
26	6 (1.0%)	0.08	0.5
27	17 (2.8%)	0.28	4.8
28	10 (1.6%)	0.03	0.3
29	5 (0.8%)	2.2	11

Table S2: Type I error rates for binary outcomes under the correlated case of 10 clusters.

Associated Cluster	MiSPU	MiRKAT	OMiAT	aMiAD	MiHC	MiAF
1	0.053	0.045	0.039	0.046	0.032	0.038
2	0.045	0.037	0.044	0.036	0.044	0.060
3	0.037	0.035	0.047	0.054	0.032	0.051
4	0.044	0.047	0.050	0.034	0.020	0.046
5	0.037	0.035	0.049	0.034	0.036	0.047
6	0.058	0.052	0.056	0.034	0.032	0.066
7	0.060	0.052	0.040	0.055	0.016	0.057
8	0.031	0.042	0.043	0.044	0.040	0.047
9	0.044	0.032	0.039	0.040	0.040	0.047
10	0.052	0.042	0.054	0.051	0.024	0.046

Table S3: Type I error rates for binary outcomes under the correlated case of 22 clusters.

Associated Cluster	MiSPU	MiRKAT	OMiAT	aMiAD	MiHC	MiAF
1	0.052	0.037	0.040	0.044	0.020	0.050
2	0.048	0.050	0.051	0.048	0.044	0.043
3	0.062	0.039	0.042	0.037	0.040	0.053
4	0.045	0.036	0.037	0.039	0.036	0.042
5	0.048	0.053	0.066	0.043	0.036	0.046
6	0.057	0.051	0.057	0.053	0.040	0.044
7	0.053	0.045	0.053	0.044	0.020	0.038
8	0.049	0.040	0.043	0.053	0.020	0.045
9	0.046	0.050	0.052	0.040	0.040	0.057
10	0.045	0.051	0.059	0.042	0.040	0.041
11	0.041	0.055	0.056	0.051	0.060	0.057
12	0.041	0.041	0.040	0.067	0.032	0.043
13	0.047	0.047	0.056	0.042	0.036	0.046
14	0.043	0.047	0.055	0.056	0.036	0.044
15	0.050	0.047	0.053	0.039	0.016	0.048
16	0.046	0.053	0.052	0.060	0.020	0.052
17	0.054	0.050	0.038	0.043	0.028	0.047
18	0.043	0.051	0.049	0.043	0.044	0.046
19	0.051	0.049	0.048	0.048	0.024	0.051
20	0.046	0.053	0.042	0.046	0.028	0.052
21	0.049	0.038	0.042	0.057	0.028	0.066
22	0.046	0.046	0.042	0.043	0.020	0.055

Table S4: Type I error rates for binary outcomes under the correlated case of 29 clusters.

Associated Cluster	MiSPU	MiRKAT	OMiAT	aMiAD	MiHC	MiAF
1	0.052	0.037	0.040	0.044	0.020	0.050
2	0.048	0.050	0.051	0.048	0.044	0.043
3	0.062	0.039	0.042	0.037	0.040	0.053
4	0.045	0.036	0.037	0.039	0.036	0.042
5	0.048	0.052	0.050	0.049	0.028	0.040
6	0.057	0.051	0.057	0.053	0.040	0.044
7	0.053	0.045	0.053	0.044	0.020	0.038
8	0.062	0.042	0.057	0.049	0.044	0.053
9	0.049	0.040	0.043	0.053	0.020	0.045
10	0.054	0.046	0.054	0.046	0.032	0.063
11	0.045	0.051	0.059	0.042	0.040	0.041
12	0.054	0.045	0.049	0.053	0.024	0.051
13	0.041	0.046	0.048	0.041	0.032	0.039
14	0.050	0.050	0.050	0.037	0.044	0.059
15	0.047	0.045	0.058	0.051	0.052	0.053
16	0.041	0.041	0.040	0.067	0.032	0.043
17	0.047	0.047	0.056	0.042	0.036	0.046
18	0.043	0.047	0.055	0.056	0.036	0.044
19	0.050	0.047	0.053	0.039	0.016	0.048
20	0.046	0.042	0.044	0.050	0.044	0.042
21	0.052	0.045	0.064	0.053	0.036	0.055
22	0.054	0.050	0.038	0.043	0.028	0.047
23	0.043	0.051	0.049	0.043	0.044	0.046
24	0.051	0.049	0.048	0.048	0.024	0.051
25	0.055	0.048	0.038	0.056	0.028	0.040
26	0.046	0.053	0.042	0.046	0.028	0.052
27	0.049	0.038	0.042	0.057	0.028	0.066
28	0.051	0.048	0.055	0.047	0.016	0.047
29	0.044	0.034	0.039	0.048	0.012	0.033

Table S5: Type I error rates for continuous outcomes under the correlated case of 10 clusters.

Associated Cluster	MiSPU	MiRKAT	OMiAT	aMiAD	MiHC	MiAF
1	0.037	0.039	0.043	0.047	0.028	0.035
2	0.031	0.039	0.050	0.048	0.036	0.043
3	0.038	0.044	0.044	0.039	0.052	0.044
4	0.050	0.047	0.036	0.060	0.020	0.052
5	0.039	0.036	0.037	0.049	0.076	0.040
6	0.047	0.051	0.051	0.049	0.052	0.039
7	0.053	0.048	0.043	0.039	0.060	0.045
8	0.032	0.035	0.031	0.053	0.032	0.037
9	0.040	0.042	0.050	0.029	0.056	0.038
10	0.062	0.066	0.054	0.038	0.044	0.043

Table S6: Type I error rates for continuous outcomes under the correlated case of 22 clusters.

Associated Cluster	MiSPU	MiRKAT	OMiAT	aMiAD	MiHC	MiAF
1	0.035	0.045	0.049	0.053	0.036	0.040
2	0.032	0.040	0.051	0.049	0.036	0.042
3	0.032	0.047	0.049	0.041	0.040	0.051
4	0.051	0.039	0.043	0.055	0.020	0.051
5	0.048	0.049	0.044	0.051	0.052	0.034
6	0.049	0.043	0.046	0.047	0.052	0.042
7	0.055	0.045	0.042	0.039	0.060	0.043
8	0.044	0.046	0.043	0.045	0.028	0.047
9	0.039	0.044	0.060	0.032	0.060	0.050
10	0.067	0.056	0.055	0.048	0.036	0.060
11	0.046	0.050	0.035	0.045	0.044	0.039
12	0.044	0.054	0.048	0.055	0.048	0.043
13	0.064	0.063	0.054	0.052	0.020	0.061
14	0.051	0.062	0.047	0.039	0.024	0.045
15	0.051	0.039	0.037	0.050	0.040	0.055
16	0.054	0.058	0.055	0.030	0.036	0.067
17	0.050	0.045	0.043	0.047	0.040	0.057
18	0.038	0.043	0.046	0.045	0.040	0.059
19	0.038	0.046	0.040	0.050	0.028	0.046
20	0.048	0.038	0.041	0.048	0.064	0.042
21	0.048	0.057	0.055	0.052	0.032	0.046
22	0.041	0.049	0.053	0.045	0.028	0.045

Table S7: Type I error rates for continuous outcomes under the correlated case of 29 clusters.

Associated Cluster	MiSPU	MiRKAT	OMiAT	aMiAD	MiHC	MiAF
1	0.035	0.045	0.049	0.053	0.036	0.040
2	0.032	0.040	0.051	0.049	0.036	0.042
3	0.032	0.047	0.049	0.041	0.040	0.051
4	0.051	0.039	0.043	0.055	0.020	0.051
5	0.032	0.043	0.043	0.048	0.028	0.037
6	0.049	0.043	0.046	0.047	0.052	0.042
7	0.055	0.045	0.042	0.039	0.060	0.043
8	0.033	0.033	0.046	0.048	0.024	0.042
9	0.044	0.046	0.043	0.045	0.028	0.047
10	0.037	0.043	0.047	0.039	0.040	0.043
11	0.067	0.056	0.055	0.048	0.036	0.060
12	0.050	0.048	0.044	0.064	0.032	0.054
13	0.039	0.039	0.036	0.049	0.072	0.038
14	0.046	0.051	0.051	0.048	0.052	0.041
15	0.056	0.050	0.048	0.044	0.056	0.041
16	0.044	0.054	0.048	0.055	0.048	0.043
17	0.064	0.063	0.054	0.052	0.020	0.061
18	0.051	0.062	0.047	0.039	0.024	0.045
19	0.051	0.039	0.037	0.050	0.040	0.055
20	0.047	0.044	0.040	0.046	0.032	0.045
21	0.044	0.051	0.054	0.033	0.060	0.044
22	0.050	0.045	0.043	0.047	0.040	0.057
23	0.038	0.043	0.046	0.045	0.040	0.059
24	0.038	0.046	0.040	0.050	0.028	0.046
25	0.063	0.068	0.066	0.039	0.036	0.036
26	0.048	0.038	0.041	0.048	0.064	0.042
27	0.048	0.057	0.055	0.052	0.032	0.046
28	0.045	0.054	0.042	0.042	0.028	0.062
29	0.048	0.057	0.046	0.050	0.040	0.040

Table S8: Sensitivity and specificity of the taxon selection algorithm for the taxa with abundance over 75%, 80% and 85% quantiles respectively under the independent case of scenario 2 with effect size 1 for continuous responses.

Quantiles	Sensitivity 75%	Specificity 75%	Sensitivity 80%	Specificity 80%	Sensitivity 85%	Specificity 85%
Cluster 1	0.441	0.865	0.467	0.870	0.500	0.857
Cluster 2	0.667	0.897	0.636	0.888	0.636	0.890
Cluster 3	0.488	0.834	0.515	0.836	0.552	0.801
Cluster 4	0.400	0.854	0.431	0.862	0.512	0.840
Cluster 5	0.313	0.950	0.366	0.951	0.483	0.955
Cluster 6	0.533	0.882	0.512	0.892	0.528	0.879
Cluster 7	0.444	0.938	0.357	0.940	0.200	0.949
Cluster 8	0.360	0.958	0.364	0.955	0.333	0.945
Cluster 9	0.417	0.929	0.410	0.930	0.457	0.928
Cluster 10	0.368	0.844	0.368	0.833	0.412	0.810

Table S9: P-values of the component tests of aMiSPU, MiRKAT, OMiAT, aMiAD and MiAF for the association test between smoking status and throat microbial community.

aMiSPU	aMiSPUu: 0.0700	aMiSPUw: 0.0015	—	—	—	—
MiRKAT	K_u : 0.0073	K_w : 0.0054	$K_{0.5}$: 0.0240	K_{BC} : 0.0210	—	—
OMiAT	aSPU: 0.0068	$\text{MiRKAT}_{\text{opt}}$: 0.0049	—	—	—	—
aMiAD	Richness: 0.3275	Shannon: 0.1132	Simpson: 0.0967	PD: 0.1806	PE: 0.0088	PQE: 0.0052
MiHC	uHC: 0.2049	wHC: 0.2220	Simes: 0.2158	—	—	—
MiAF	MiAF_{u}: 0.128	MiAF_{w}: 0.0023	$\text{MiAF}_{0.5}$: 0.005	MiAF_{a}: 0.0013	—	—

Table S10: P-values of the component tests of aMiSPU, MiRKAT, OMiAT, aMiAD and MiAF for the association test between HIV infectious status and gut microbial community.

aMiSPU	aMiSPUu: 0.4457	aMiSPUw: 0.0047	—	—	—	—
MiRKAT	K_u : 0.0001	K_w : 0.0013	$K_{0.5}$: 0.0001	K_{BC} : 0.0001	—	—
OMiAT	aSPU: 0.0001	$\text{MiRKAT}_{\text{opt}}$: 0.0001	—	—	—	—
aMiAD	Richness: 0.0249	Shannon: 0.1877	Simpson: 0.3427	PD: 0.2425	PE: 0.0125	PQE: 0.0002
MiHC	uHC: 0.0001	wHC: 0.0001	Simes: 0.4680	—	—	—
MiAF	MiAF_{u}: 0.0224	MiAF_{w}: 0.0046	$\text{MiAF}_{0.5}$: 0.0002	MiAF_{a}: 0.0001	—	—

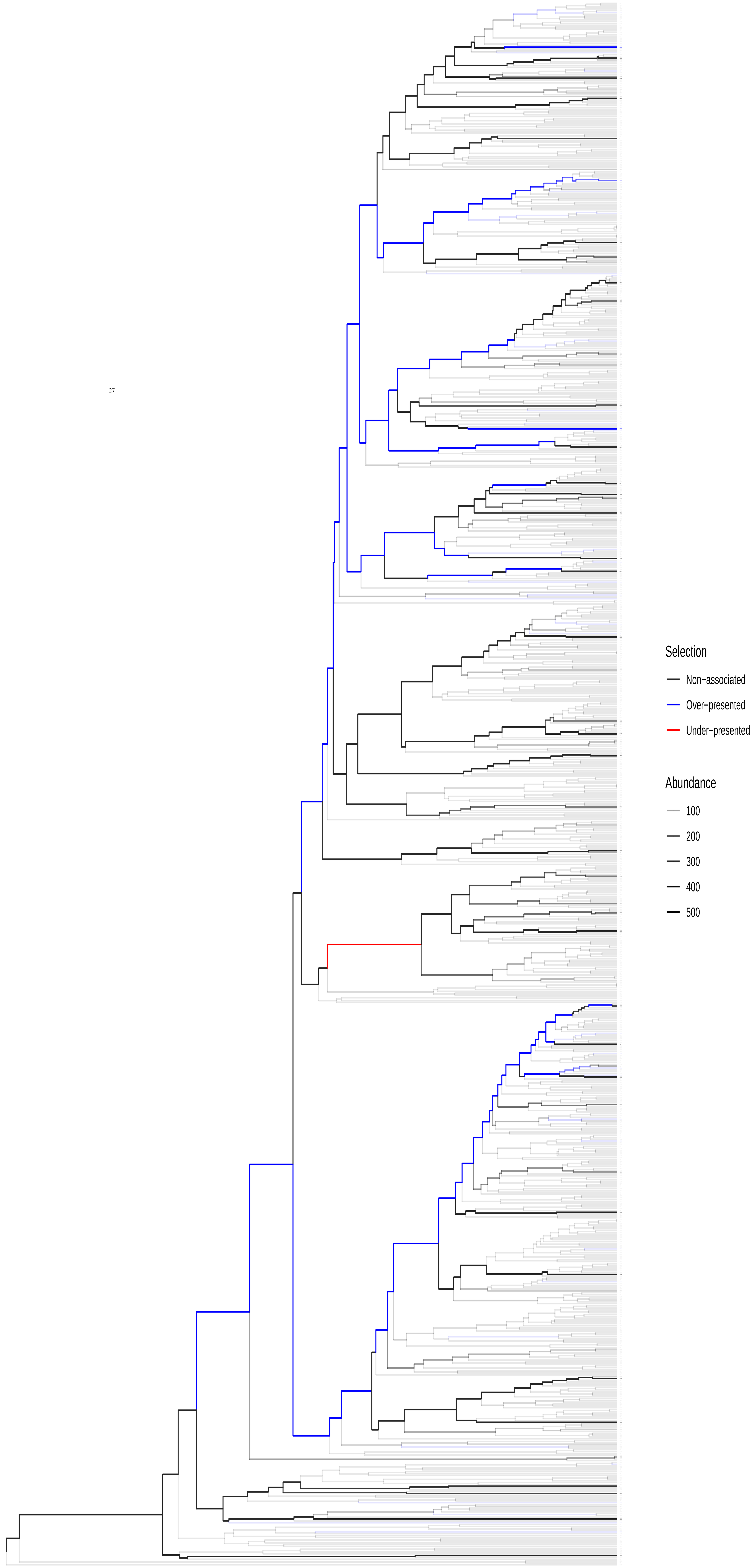

Figure S17: Taxon selection in the phylogenetic tree for a throat microbiome dataset. Grey edges stand for non-associated signals, while blue and red edges represent associated signals. The blue and red ones were selected from lower and upper one-sided p-values respectively. The tip label in R package MiSPU is shown on the right side.
